# Hans Asperger, National Socialism, and “race hygiene” in Nazi-era Vienna

**DOI:** 10.1186/s13229-018-0208-6

**Published:** 2018-04-19

**Authors:** Herwig Czech

**Affiliations:** Ethics, Collections, and History of Medicine, Medical University of Vienna, Währinger Straße 25, 1090 Vienna, Austria

**Keywords:** Hans Asperger, Biography, Asperger’s syndrome, Therapeutic pedagogy (Heilpädagogik), Autism, World War II, National Socialism, History, Vienna/Austria, Child psychiatry, Pediatrics

## Abstract

**Background:**

Hans Asperger (1906–1980) first designated a group of children with distinct psychological characteristics as ‘autistic psychopaths’ in 1938, several years before Leo Kanner’s famous 1943 paper on autism. In 1944, Asperger published a comprehensive study on the topic (submitted to Vienna University in 1942 as his postdoctoral thesis), which would only find international acknowledgement in the 1980s. From then on, the eponym ‘Asperger’s syndrome’ increasingly gained currency in recognition of his outstanding contribution to the conceptualization of the condition. At the time, the fact that Asperger had spent pivotal years of his career in Nazi Vienna caused some controversy regarding his potential ties to National Socialism and its race hygiene policies. Documentary evidence was scarce, however, and over time a narrative of Asperger as an active opponent of National Socialism took hold. The main goal of this paper is to re-evaluate this narrative, which is based to a large extent on statements made by Asperger himself and on a small segment of his published work.

**Methods:**

Drawing on a vast array of contemporary publications and previously unexplored archival documents (including Asperger’s personnel files and the clinical assessments he wrote on his patients), this paper offers a critical examination of Asperger’s life, politics, and career before and during the Nazi period in Austria.

**Results:**

Asperger managed to accommodate himself to the Nazi regime and was rewarded for his affirmations of loyalty with career opportunities. He joined several organizations affiliated with the NSDAP (although not the Nazi party itself), publicly legitimized race hygiene policies including forced sterilizations and, on several occasions, actively cooperated with the child ‘euthanasia’ program. The language he employed to diagnose his patients was often remarkably harsh (even in comparison with assessments written by the staff at Vienna’s notorious Spiegelgrund ‘euthanasia’ institution), belying the notion that he tried to protect the children under his care by embellishing their diagnoses.

**Conclusion:**

The narrative of Asperger as a principled opponent of National Socialism and a courageous defender of his patients against Nazi ‘euthanasia’ and other race hygiene measures does not hold up in the face of the historical evidence. What emerges is a much more problematic role played by this pioneer of autism research. Future use of the eponym should reflect the troubling context of its origins in Nazi-era Vienna.

## Background

**Fig. 1:**
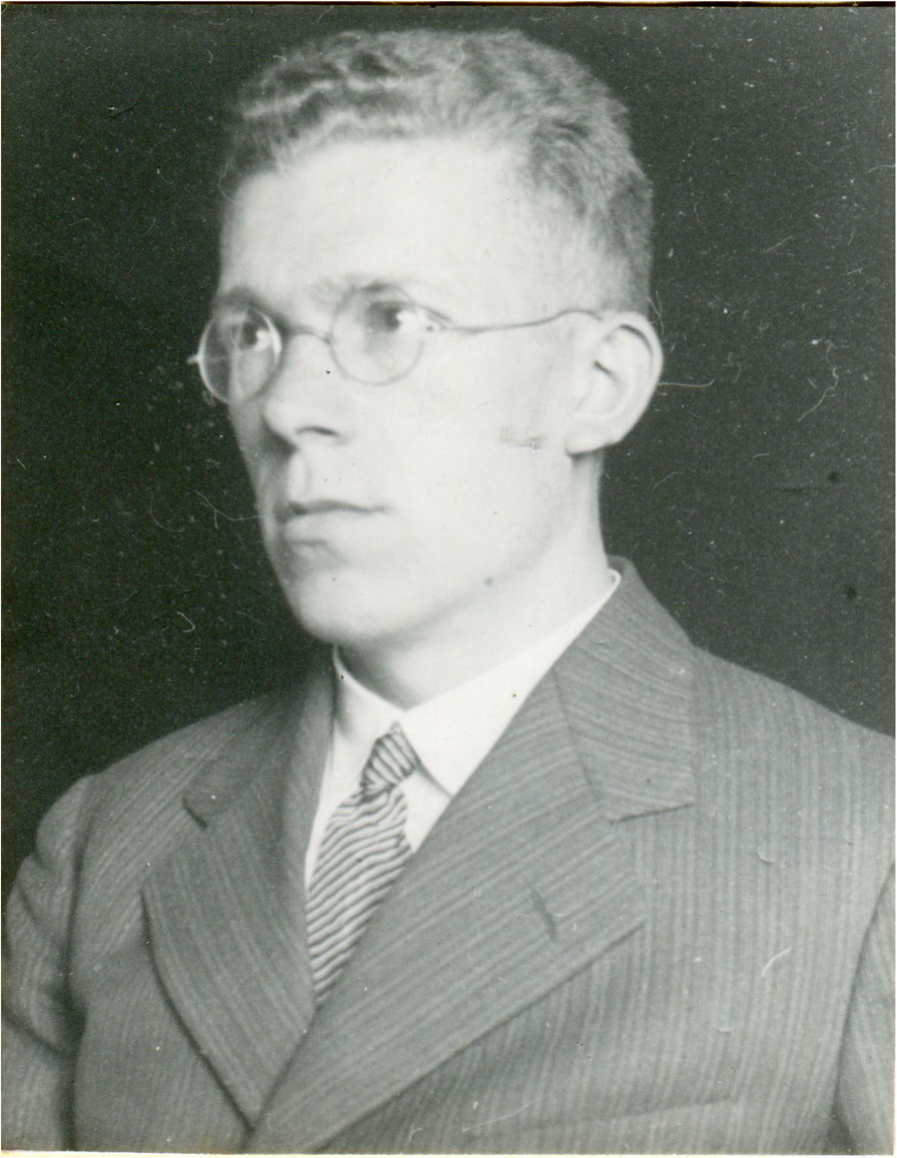
Portrait of Hans Asperger (1906–1980) from his personnel file, ca. 1940 (WStLA, 1.3.2.202.A5, Personalakt)

Despite the international prominence of Hans Asperger (Fig. 1) as one of the pioneers in the history of autism and as the namesake of Asperger’s syndrome, factual knowledge about his life and career is limited. This is surprising given that his successful career in Nazi-controlled Vienna raises questions concerning his potential political or professional involvement with National Socialism. The existing literature on the topic has tended to downplay or overlook any such involvement, or even to postulate that Asperger took a position of active resistance. With few exceptions, however, these judgments are based on a limited number of sources—a few passages from Asperger’s Nazi-era publications, particularly a 1938 lecture containing the first references to “autistic psychopaths” [1] and his 1944 postdoctoral thesis [2],^1^ and statements by Asperger himself or by persons close to him from after 1945 (most importantly, a 1974 radio interview [3]).

The goals of this paper, based on comprehensive archival research, are to provide an account of Asperger’s life and career during National Socialism and to submit prevailing narratives to the test of historical evidence. The picture that emerges is that of a man who managed to further his career under the Nazi regime, despite his apparent political and ideological distance from it. This was not least due to opportunities created by the political upheaval after Austria’s *Anschluss* (annexation) to Germany in 1938, including the expulsion of Jewish physicians from the profession. (On the expulsion of Jews from the university clinic, which began before 1938, see [4] and below). As I will demonstrate, this career was made possible by Asperger’s political concessions to the Nazi ideology and involved a certain degree of collaboration with the race hygiene apparatus, including the Nazis’ child “euthanasia” program.

The analysis of patient case files written by Asperger and his colleagues from 1928 to 1944—a crucial set of documents mistakenly assumed to have been destroyed in World War II—sheds new light on the fate of Asperger’s patients during the Nazi period (on Asperger’s case files, see the “Asperger’s Jewish patients” to “Asperger’s diagnoses compared to those at Spiegelgrund” sections).

A review of the existing literature on Asperger’s life and career shows the current fault lines in the narrative of his Nazi-era trajectory. Lorna Wing’s seminal paper from 1981 which popularized the term “Asperger’s syndrome” made no reference to the historical context of Asperger’s work [5]. Similarly, Uta Frith’s 1991 book chapter “Asperger and his syndrome” barely mentioned National Socialism in the few pages dedicated to Asperger’s professional and personal life in Vienna during the 1930s and 1940s. Based on her reading of Asperger’s 1944 article on “autistic psychopaths,” she stated that “Asperger clearly cared about these children, who in most people’s eyes were simply obnoxious brats” ([6]: 7). Her text established what has become the most common view of Asperger’s behavior during the Nazi period, namely that he defended his patients against the Nazi regime at great personal risk: “Far from despising the misfits, he devoted himself to their cause—and this at a time when allegiance to misfits was nothing less than dangerous.” She defended Asperger against accusations of “allegiance to Nazi ideology” that had been raised because of his early commitment to the German Youth Movement ([6]: 10). Eric Schopler, one of Asperger’s fiercest critics, was one of those who explicitly drew this connection, but apparently had no evidence to back his accusations.^2^ When Frith published an annotated translation of Asperger’s 1944 paper, her sole comment on its origin in Nazi-era Vienna was that it contained only one reference to “fascist ideology at a time when it would have been opportune to make many more such references” ([7]: 86).^3^

Brita Schirmer published the first paper explicitly addressing Asperger’s role during National Socialism [8]; her stance is already indicated in the subtitle: “Hans Asperger’s defense of the ‘autistic psychopaths’ against Nazi eugenics.” Her argument was based on Asperger’s 1938 paper “The mentally abnormal child” [1] from which she drew conclusions similar to Uta Frith’s. A 2003 paper by Helmut Gröger, also in German, examined possible influences of Nazi race ideology on Asperger’s published work. Citing no less than 23 of Asperger’s publications in the years from 1937 to 1974, Gröger concluded that Asperger generally “avoided topics touching race ideology” and maintained a “critical, differentiated attitude” ([9]: 204, 206).^4^ In line with the other authors cited here, Gröger credited Asperger with advocating on behalf of his patients, defending their value as human beings, and calling for loving care for each of them ([9]: 204–5, 210).

Interestingly, Gröger mentioned—without discussing the implications—that Asperger’s name “appears” in the files of a 3-year-old girl with mental deficiencies who was sent to the child “euthanasia” clinic Am Spiegelgrund in Vienna ([9]: 209). As I demonstrate in the “Limits of ‘educability’: Asperger and the Spiegelgrund ‘euthanasia’ facility” section, Herta Schreiber, the girl in question, was in fact transferred to the Spiegelgrund facility on Asperger’s authority and died there 2 months later.

From 2005, cracks started to appear in the predominantly apologetic narrative of Asperger’s role during National Socialism. Michael Hubenstorf, in an extensive book chapter on the history of Vienna University’s Pediatric Clinic where Asperger worked, presented a host of previously unknown aspects of Asperger’s career. The close ties between the pediatric clinic and the “euthanasia” facility Am Spiegelgrund, including connections between Asperger and Spiegelgrund’s director Erwin Jekelius (1905–1952, Fig. 2), are of particular importance in the context of this paper ([4]: 171–4). Hubenstorf also documented the relationship between Asperger and his mentor Franz Hamburger, a fervent Nazi ideologue ([4]: 93, 118–9, 126–35, 191–3; see the “‘The best service to our *Volk*’: Asperger and Nazi race hygiene” to “Asperger’s diagnoses compared to those at Spiegelgrund” sections). Based among other sources on Hubenstorf’s work, on personal documents, and on her own memories, Maria Asperger Felder published a nuanced portrait of her father, not shying away from his possible involvement in National Socialism—without, however, adding significant new facts [10]. Citing Schirmer [8], Daniel Kondziella in a 2009 paper on 30 neurological eponyms associated with the Nazi era included Asperger among the “physicians with ambivalent roles” because he had “been accused on uncertain grounds of harboring sympathy for Nazi politics” (while he had also “cautiously defended mentally disabled children”) ([11]: 59).

**Fig. 2:**
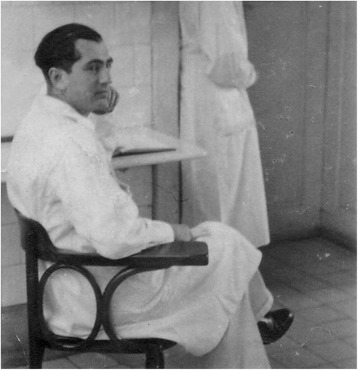
Asperger’s colleague Erwin Jekelius, who during the Nazi period became director of the Spiegelgrund child “euthanasia” clinic and coordinator of the “T4” killing program in Vienna (DÖW)

Some preliminary results of my own research were presented at a 2010 symposium marking the 30th anniversary of Asperger’s death and published in the conference proceedings ([12]; see also [13]: 201, 206, 217). In the same volume, Helmut Gröger argued along the lines of his above-cited 2003 article [14], while Roxane Sousek hinted at problematic aspects of Asperger’s activities ([15]: 19). Ina Friedmann in her recent work on the topic also refrained from presenting an idealized picture of Asperger and the Austrian school of Heilpädagogik (therapeutic pedagogy) [16–18].

While evidence for problematic aspects of Asperger’s career have thus begun to emerge in German-language publications, authors in the English-speaking world often continued to perpetuate a predominantly apologetic narrative based on the limited range of sources available to them. In 2007, a letter to the editors in one of the leading autism journals claimed that Asperger “tried to protect these children from being sent to concentration camps during World War II,” a statement that is confusing at best, since child “euthanasia” had nothing to do with concentration camps ([19]: 2020).^5^

Adam Feinstein’s 2010 book on the history of autism illustrated the increasing gap between the English- and German-language literature. The author qualified the affirmative references to Nazi ideology in some of Asperger’s papers as a deliberate tactic to deceive “the Nazis” about his true intentions, namely to protect his patients. A cornerstone of his argument is Asperger’s claim that he had faced arrest by the Gestapo for his stance against Nazi race hygiene policies ([20]: 15–18). Steve Silberman’s 2015 book *NeuroTribes*, written for a general audience, also pushed the narrative of Asperger as an Oskar Schindler-like protector of children with autism. One of Asperger’s alleged strategies was that he “intentionally highlighted his ‘most promising’ cases to deflect the wrath of the Nazis” ([21]: 216). As far as Asperger’s conduct during National Socialism is concerned, Silberman’s argument (and the evidence presented) is very similar to Adam Feinstein’s and some of the other texts already mentioned ([21]: 108–9, 128–9, 137–8). The “‘The best service to our *Volk*’: Asperger and Nazi race hygiene” section is dedicated to a discussion of these and similar claims.

One of Silberman’s original findings regards the question of Asperger’s and Kanner’s respective roles in the “discovery” of autism. Georg Frankl (1897–1976), a close collaborator of Asperger’s, left Vienna for the USA in 1937, going on to work with Leo Kanner ([21]: especially 167–8, 180). This information revived earlier suspicions that Kanner had known about Asperger’s work before his own publications on the topic, based on the fact that Asperger had already mentioned autistic psychopathy in a 1938 publication [1], years before his more widely known postdoctoral thesis [2], and that the Austrian-born Kanner had access to German-language medical publications [22, 23].^6^

The latest addition to a growing body of literature on the subject, John Donvan and Caren Zucker’s *In a Different Key* is the first English-language publication to break with the narrative of Asperger as an active opponent to Nazi race hygiene and to introduce critical, hitherto unknown elements into the debate on his Nazi-era trajectory. This shift is mainly based on sources that I shared with the authors, which are presented in detail below ([24]: 316–41).^7^

Although the precise nature of Asperger’s relationship to National Socialism has been the elephant in the room for some time now, the necessary questions have so far evidently either not been asked at all, or they have been answered on the basis of a too limited number of sources. In what follows, I will present a more multifaceted picture both of Asperger’s Nazi-era career and of the historical context of the inception of autism, based on an extensive body of sources, many of which are presented here for the first time.

## Methods

This paper is based on a qualitative analysis of documents relating to Hans Asperger’s life, work, and political orientation from archives in Austria and (to a lesser degree) Germany, and of his own publications, most of which have not previously been examined with regard to the questions raised here. The documentary sources include, among others, Asperger’s personnel files, political assessments by Nazi authorities, and medical case records from various institutions, most importantly from the child “euthanasia” clinic Am Spiegelgrund and Asperger’s Heilpädagogik ward. Despite claims to the contrary ([21]: 140, [25]: 37, [26]: 22), these records were not destroyed in the war. Apart from a gap between 1945 and 1969, the files (which go back as far as 1912) are today kept in the Municipal and Provincial Archives of Vienna.^8^ They pertain to those children admitted as inpatients; the documentation on the much larger number of children examined at the outpatient clinic is lost. From the critical years of 1938 to 1944, 1012 case files survive. Between 1940 and 1944, 62.7% of patients admitted were boys and 37.3% girls. Apart from a number of recurring elements (such as admission forms), the files vary in content and scope. It cannot be ruled out that single documents or whole files have been lost or purged. These records are analyzed here for the first time.

## Results and discussion

### Asperger’s career before 1938

In 1911, Erwin Lazar (1877–1932) established the *Heilpädagogische Station* (Therapeutic Pedagogy Ward) at the Vienna University Children’s Clinic (part of the city’s general hospital), which had achieved international renown under its director Clemens von Pirquet (1874–1929) ([27]: 320, [28]: 161).^9^ Lazar regarded Heilpädagogik as a direct descendant of psychiatry, although the classic psychiatric illnesses such as psychoses were rarely diagnosed in the children he treated. Instead, he diagnosed the vast majority of his patients with “psychopathy” or mental “imbalance.” Most of the ward’s patients—in 1925, he mentioned a figure of 5000 annually—were diagnosed at the outpatient clinic. Only a relatively small number—complicated cases or cases of special clinical interest—was admitted over longer periods. Many children were referred by welfare institutions, the police, or the courts. Under Lazar, Heilpädagogik took inspiration from a variety of concepts, including Cesare Lombroso’s criminal biology, Ernst Kretschmer’s constitutional types, and Sigmund Freud’s psychoanalysis [28].

Asperger joined the children’s clinic in May 1931 under Pirquet’s successor Franz Hamburger (1874–1954). In 1932, he started working at the clinic’s Heilpädagogik ward as an “auxiliary physician” (*Hilfsarzt*). In May 1935, he took charge of the ward and reached the position of an assistant.^10^ Asperger had not obtained his specialist doctor qualification in pediatrics and had published only a single work in Heilpädagogik (on bed-wetting) [29].^11^ This raises the question why Asperger’s colleague Georg Frankl was not promoted to the position—Frankl was 9 years older and had been working at the ward since 1927.^12^ Two years after Asperger’s promotion, Frankl emigrated to the USA, where he joined Leo Kanner at Johns Hopkins ([21]: 122).^13^ Another highly qualified Jewish employee, the psychologist Anni Weiss (1897–1991), who later married Frankl, had already left Austria in 1935 ([21]: 122).^14^

Austrian universities were sites of virulent anti-Jewish agitation at the time (see [30]), which almost certainly was a factor in their decision to leave. Jewish doctors faced increasing difficulties in securing university positions, with some clinics and departments practically closed to Jews ([31]: 312). With Hamburger’s appointment as chair in 1930, the children’s clinic became a flagship of anti-Jewish policies long before the Nazi takeover ([4]: 69, 112). Regarding Anni Weiss and Valerie Bruck (1894–1961), Asperger’s immediate predecessor as head of the Heilpädagogik ward, hostility towards working women also played a role: The Austrofascist regime (1933–1938) sought to push women out of the labor market, a stance shared by Nazi ideologues such as Hamburger ([16]: 181).^15^

After Pirquet’s sudden death in 1929, Hamburger introduced sweeping changes at the clinic. Pirquet’s former collaborators, many of them Jewish, were replaced. The political orientation of Hamburger’s assistants is illustrated by the fact that of those who attained the highest academic qualification (*Habilitation*), all but one were dismissed in 1945 as Nazis—the exception being Hans Asperger ([27]: 320).^16^ Among Hamburger’s recruits was Erwin Jekelius, who later became responsible for the deaths of thousands of psychiatric patients and mentally disabled children. He remained at the clinic from August 1933 to February 1936, spending part of this time at the Heilpädagogik ward.^17^ Another result of Hamburger’s influence was a sharp decline in scientific standards and output ([4]: 87–94, 104, 117–8).

Hamburger and Jekelius were not the only fervent Nazis with whom Asperger had close professional contact during his early career. In 1932, he co-authored a paper with Erwin Risak (1899–1968), who had been his colleague at the university’s III. Medical Clinic for a few weeks in 1931 [32].^18^ Under Franz Chvostek junior (1864–1944), this clinic became known as a hotbed of Pan-German nationalist and Nazi agitation. Risak became an assistant to Hans Eppinger junior (1879–1946), director of the I. Medical Clinic, who was later involved in the Dachau seawater experiments.^19^ Following the Anschluss, Risak became one of the figureheads of the Nazi Party (NSDAP) in the Vienna Medical Faculty, along with figures such as Hamburger, the anatomist Eduard Pernkopf (1888–1955), and others ([4]: 129).

Whatever the specific motivations for Hamburger’s decision to appoint Asperger as the head of the Heilpädagogik ward in 1935, Asperger’s promotion was aided by the anti-Jewish and misogynist tendencies then dominating Austria’s social and political life. Although Asperger did not join the Nazis, due to his Pan-Germanic, *völkisch* orientation, he shared considerable ideological common ground with Hamburger and his network, allowing him to blend in without apparent frictions. When anti-Jewish persecution became state policy after the Anschluss, 65% of Viennese physicians were classed as Jewish according to the Nuremberg Laws, including 77 pediatricians (70% of the specialists in this field). Tellingly, in 1938, not one Jewish pediatrician who had attained a *Habilitation* was working in Hamburger’s clinic ([4]: 71–3, 112).^20^

Asperger had the unreserved support of Franz Hamburger, even if he did not belong to his mentor’s circle of underground Nazi activists. At a young age, and in an environment marked by political strife and a difficult labor market, he rose to Austria’s most prominent position in the expanding field of Heilpädagogik, which would soon be forced to find its place within the new order of the Nazi state.

### Asperger’s political background before 1938

In order to understand how Asperger positioned himself vis-à-vis the Nazi regime after March 1938, it is first necessary to examine his political orientation during his formative years, when there was still a spectrum of political options to choose from. This will help explain why Asperger in 1938 found enough common ground with National Socialism to establish himself as a credible fellow traveler in the eyes of the party, without directly embracing National Socialism.

In Asperger’s own words, his formative experience within the polarized political landscape of interwar Austria was membership in the so-called *Bund Neuland*, a Catholic youth organization focused on outdoor activities, with roots in the predominantly *völkisch*-nationalist *Wandervogel* and the German Youth Movement ([4]: 192–3). In 1914, 92% of the Wandervogel chapters (in Germany and Austria) had no Jewish members, due mostly to formal anti-Jewish regulations ([33]: 92–4).

Founded in 1921, the Austria-based Bund was a split-off from the Christian-Social Student Union (CDSB) but stressed its affinities with the German Youth Movement as represented by the “Meißner formula,” which Asperger cited in 1974 as a guiding principle in his life [3].^21^ After World War I, the CDSB had become rife with aggressive anti-Jewish propaganda, including calls to boycott Jewish businesses ([33]: 175–81), tendencies which the Bund shared.

The Bund’s intellectual influence was greater than its approximately 2000 strong membership would suggest ([34]: 92).^22^ It defined itself as Christian, Catholic, and Pan-German, and in sharp opposition to everything perceived as Marxist-leftist, liberal, or modern, which included parliamentary democracy.^23^ There was a degree of political diversity, and the Bund is sometimes classed as a “socially progressive” Catholic organization because some members supported social reforms in order to bring workers into the fold of the Church ([35]: 46). Nevertheless, in its fundamental principles, the Bund stood close to the fascist and authoritarian currents of the time ([36]: 835). A draft program from 1931 confirmed its opposition to the democratic state “in its current form” and stated that “the equivalence between *Volk* and state leads necessarily to the ideal of the Greater German Reich” (cited in [34]: 99).

During the 1930s, important sections of the Bund were infiltrated by Hitler Youth groups and members of other Nazi organizations ([34]: 95, 193, [37]). In 1935/1936, press reports estimated that 20% of Neuland members were (illegal) Nazis ([38]: 70–1). The authoritative account of its history states that the “predominant majority in the Bund was oriented towards Pan-Germanism, supported Austria’s unification with Germany, and was at best indifferent vis-à-vis National Socialism,” despite the fact that the official policy was to identify and exclude Hitler Youth cells within the organization ([36]: 586–7). The most striking example for the Bund’s infiltration is its leader Anton Böhm (1904–1998), who joined the NSDAP in 1933 and remained an “illegal” party member until the Anschluss in 1938, even serving as an informant to the Austrian Nazis’ intelligence services and the Gestapo in Munich ([34]: 103, 189–95).^24^ Arguably, the Bund constituted one of Nazism’s most important intellectual bridgeheads in the powerful Austrian Catholic milieu during the crucial years leading up to the Anschluss ([34]: 100–1).

In 1933, Böhm published a programmatic comment on the political situation following the Nazi takeover in Germany, referring also to the persecution of the Jewish population: “There can be no doubt that the strong Jewish influence in Germany has had baleful consequences. Therefore, the anti-Jewish measures in Germany are justified as acts of national self-defense” ([39]: 106–7).

Over the following years, the Bund published a number of articles supporting the anti-Jewish persecutions in Nazi Germany [40].^25^ The Bund’s official mouthpiece also denounced the Viennese “Jewish press” as a corrosive influence in Austrian public life, attacked Jews as an alien element within the Catholic-German Austrian population, and warned against the dangers of “racial” and religious intermarriage ([41–43]: 20–1, [44]: 215).

While the Bund’s periodical welcomed the German Nazis’ anti-Jewish policies, its stance towards National Socialism as a whole was more complex. Although the Bund shared the Nazis’ contempt for parliamentary democracy and all forms of cultural and intellectual modernism, as well as their glorification of the German *Volk* as the basis for cultural regeneration, they nevertheless regarded the NSDAP with the same suspicion as they did all other political parties. Catholicism remained the central point of reference, and the NSDAP was mainly judged according to its policies towards the Church. In 1933, Böhm signaled that the organization would support the Nazis’ “national revolution” in Germany provided that Hitler would choose to strengthen the anti-capitalist tendencies of his movement and, more importantly, grant the Catholic Church and Christianity generally its due place in the German Reich [45]. In the following issue, Böhm openly called for the integration of the Nazis into the Austrian government ([39]: 110).

Within the organization, which was far from ideologically homogenous, Asperger belonged to a group called the *Fahrende Scholaren* (Wandering Scholars), part of the Bund’s decidedly *völkisch* and right-wing faction. He was associated with the inner circle of “organic romanticists” around Michael Pfliegler (1891–1972), a Catholic priest and founding member of the Bund, and its leader Anton Böhm ([33]: 207–8, 342; [34]: 63–5).^26^

After the Anschluss, at least some of the Bund’s former members joined anti-Nazi networks, notably in Innsbruck and Lower Austria ([35]: 46). Resistance activities, which included an assembly of 300 youths in Vienna on the night of the German invasion, were primarily the work of the younger generation. By contrast, the older generation to which Asperger belonged tended to seek immediate accommodation with Nazism ([36]: 586–8, 839). This is evident in Asperger’s path after 1938, as he joined a number of Nazi organizations (although not the NSDAP) and sought to accommodate himself with the new regime.

Bund Neuland was the most important but not the only political influence in Asperger’s life. The physicians within the Bund delegated him to the St. Lukas guild, which promoted medical ethics along Catholic principles. Regarding eugenics, its position was ambivalent; it opposed some tenets of Nazi race hygiene such as forced sterilizations while developing its own eugenic program within the bounds of Catholicism ([46]: 106–16).^27^

**Fig. 3:**
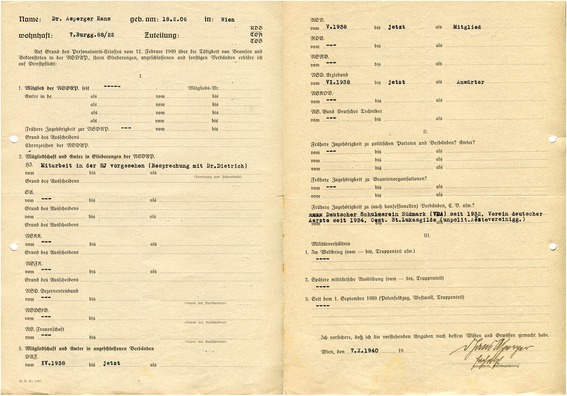
In this questionnaire from October 1940, Asperger reported several memberships in organizations affiliated with the Nazi Party. He refrained, however, from joining the NSDAP itself (WStLA, 1.3.2.202.A5, Personalakt)

According to a questionnaire dated 1940, Asperger was also a member of the *Verein Deutscher Ärzte in Österreich* (Association of German Doctors in Austria, Fig. 3).^28^ “German” in this context refers to a Pan-German orientation, excluding Jewish doctors. The *Verein Deutscher Ärzte* emerged from a 1904 federation between the anti-Jewish *Verein Wiener Ärzte* and various Pan-German medical associations in Austria ([4]: 78). In the 1920s and again after the Nazi takeover in Germany, the organization called to limit the number of Jewish students ([47]: 90). A considerable portion of leading (non-Jewish) Viennese doctors, including the former head of the pediatric clinic Clemens Pirquet, belonged to the association—an indication of how widespread anti-Jewish sentiment was in Viennese medical circles ([4]: 78).

In the same questionnaire, Asperger mentioned another membership indicating his affinity to the Pan-German nationalist wing, despite his Catholic orientation. In 1932, he joined the *Deutscher Schulverein Südmark* (German School Association for the Southern Border Region), which sought to strengthen German cultural influence abroad with the help of German-speaking minorities. Many of the *Schulverein*’s members were close to the Austrian *Großdeutsche Volkspartei* (Greater German People’s Party), which in 1933 formed an alliance with the Austrian Nazi Party.^29^

Despite these associations, there is no indication that Asperger actively sympathized with the Nazi movement prior to 1938, unlike many of his colleagues. Rather, the evidence points to an ambivalent attitude. Potential obstacles to his supporting National Socialism were his religious convictions, his humanist background, and his elitist, cultivated habitus. Furthermore, following the ban of the Austrian Nazi Party in 1933, the movement remained attractive only to a core of ideologically hardened supporters, whereas for mere sympathizers or opportunists, the risks of adherence far outweighed potential advantages. Nevertheless, Asperger’s record of organizational affiliations over the years prior to 1938 suggests that there was more common ideological ground than has previously been acknowledged. Asperger’s political socialization in Neuland likely blinded him to National Socialism’s destructive character due to an affinity with core ideological elements (see [40]: 848–9).^30^ In 1974, Asperger himself put it this way: “[Then] the National Socialist time arrived, whereupon it was clear from my previous life that one could well go along with many let’s say quote unquote ‘national’ things, but not with the inhuman [ones]” [3].^31^

In post-World War II Austria, “national” as a political label invariably referred to Pan-Germanism and is to this day used by right-wing groups as a euphemism to avoid overt association with (neo-) Nazism. In other words, Asperger in 1974 distanced himself from the *Unmenschlichkeiten* (inhumanities) of National Socialism, but not from its Pan-German program, which in 1938 had led to the annexation of Austria and later to World War II. Asperger’s ambivalent attitude towards National Socialism was already palpable in a diary entry from April 1934 (when he spent some time in Nazi Germany), which evinced both skeptical distance and a certain fascination: “How a whole people marches in one direction, fanatical, with narrowed vision, certainly, but with enthusiasm and dedication, with enormous discipline and formidable vigor. Only soldiers, soldierly thinking—ethos—German paganism.”^32^

### Political trajectory after the Anschluss in 1938

The established narrative concerning Asperger’s relationship to National Socialism after 1938 is that Asperger actively opposed the regime, or at least kept his distance, under considerable professional and personal risk. In 1993, Lorna Wing argued that as a devout Catholic, he could not have been a Nazi ([24]: 330). This argument is misleading, however, given the overlap discussed above between Catholicism and the *völkisch* extreme right represented by organizations such as the Bund Neuland.

The strongest claim to the effect that Asperger was an active opponent of the Nazis and that he risked his life defending the children in his care is based on an episode reported in Adam Feinstein’s book on the pioneers of autism research. Allegedly, the Gestapo twice came to the clinic to arrest Asperger, either because of his 1938 talk [1] or because he had refused to “hand [patients] over to officials” ([20]: 17–8). The only known source for this claim is Asperger himself, who mentioned the incident in 1962 at his inauguration as the Vienna chair of pediatrics [48] and in the above-cited 1974 interview:

It is totally inhuman—as we saw with dreadful consequences—when people accept the concept of a worthless life. […] As I was never willing to accept this concept—in other words, to notify the [Public] Health Office of the mentally deficient—this was a truly dangerous situation for me. I must give great credit to my mentor Hamburger, because although he was a convinced National Socialist, he saved me twice from the Gestapo with strong, personal commitment. He knew my attitude but he protected me with his whole being, and for that I have the greatest appreciation [3].^33^

This is the only recorded instance I could find in which Asperger publicly mentioned Nazi “euthanasia”—despite the fact that this was such an incisive event for his field and its patients.^34^ According to this account, the Gestapo was after Asperger because he refused to report patients with certain deficiencies to Vienna’s Public Health Office. It is true that doctors were increasingly obliged to report patients to the authorities in defiance of patient/physician confidentiality. Regarding race hygiene policies, the two most important instances were the compulsory reporting of patients as mandated by the sterilization law and of children with mental deficiencies who were slated for “euthanasia.”^35^ Based on the available evidence, it is impossible to determine whether Asperger in some cases abstained from reporting children who met the criteria for child “euthanasia.” However, it is documented that he personally referred a number of children to the Spiegelgrund “euthanasia” facility (“Limits of ‘educability’: Asperger and the Spiegelgrund ‘euthanasia’ facility” and “Asperger’s diagnoses compared to those at Spiegelgrund” sections).

Other facts speak against Asperger’s self-portrayal as a man persecuted by the Gestapo for his resistance to Nazi race hygiene, who had to flee into military service to avoid further problems. On several occasions, he published approving comments on race hygiene measures such as forced sterilizations (see [49]: 353; for further examples, see the “‘The best service to our *Volk*’: Asperger and Nazi race hygiene” section), and as is discussed further below, the Nazi hierarchy saw him as someone willing to go along with race hygiene policies. In July 1940, the deputy Gauleiter of Vienna wrote to Asperger’s superior and protector Franz Hamburger that the party had “no objections whatsoever” against his assistant.^36^ The Vienna Gestapo, when asked for a political assessment of Asperger, answered in November 1940 that they had nothing on him.^37^ This contradicts claims that Asperger’s early publications after the Anschluss, including those most frequently cited as proof for his public opposition to Nazi policies, were perceived by the regime as expressions of political opposition.

**Fig. 4:**
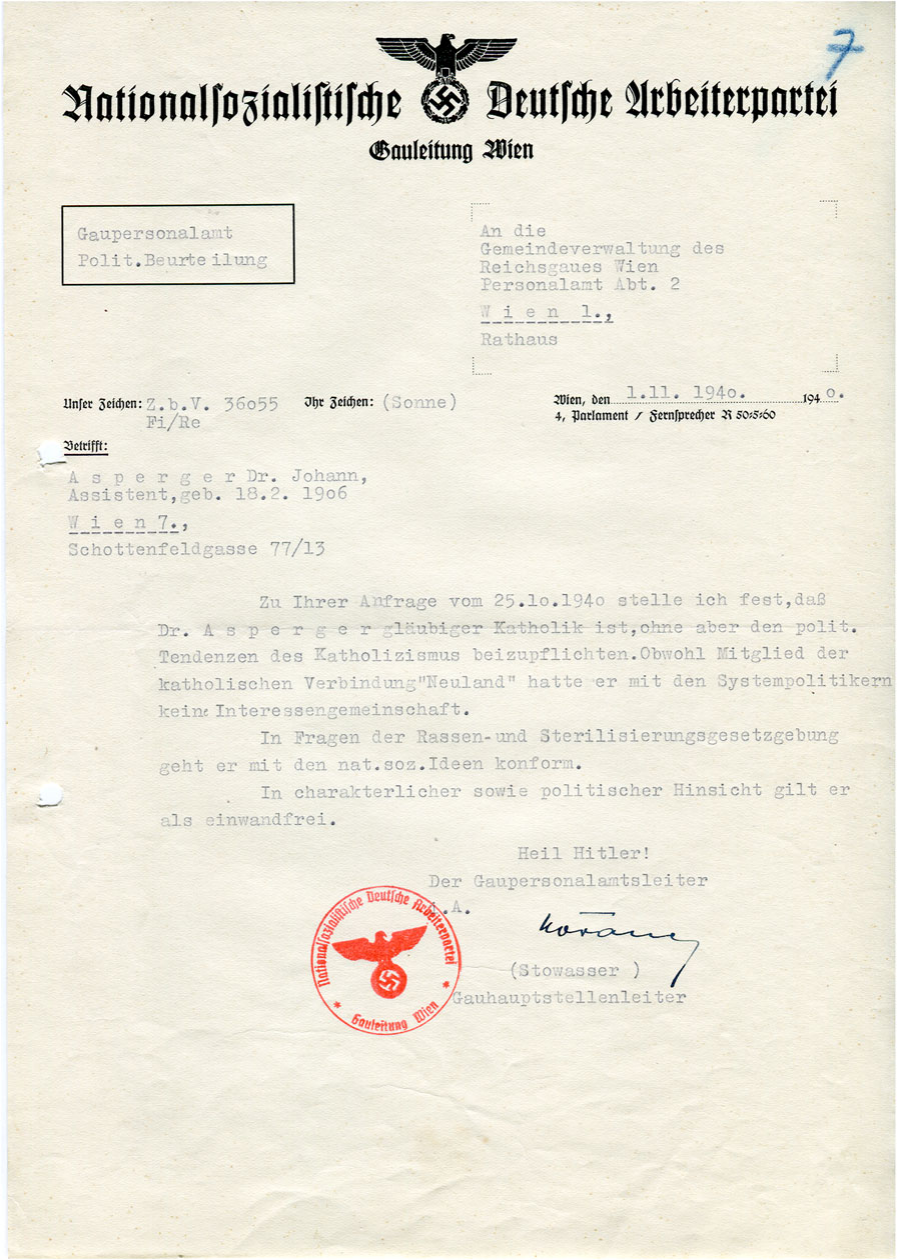
Despite Asperger’s Catholic orientation, the Nazi Party authorities considered Asperger to be “politically irreproachable” and as someone who “was in conformity with the National Socialist racial and sterilization laws” (WStLA, 1.3.2.202.A5, Personalakt)

Initially, before Asperger had a chance to prove his willingness to adjust to the new political order, the NSDAP was unsure about his loyalty. Immediately following the Anschluss, a preliminary investigation was initiated to decide whether the “Decree for the Reorganization of the Austrian Professional Civil Service” of 31 May 1938, which stipulated the dismissal of Jewish and politically undesirable officials ([50]: 235), applied to Asperger. In June 1939, the official charged with implementing the decree, Otto Wächter (1901–1949), decided to close the file because Asperger was “politically acceptable” from the National Socialist standpoint.^38^ According to the Vienna NSDAP Personnel Office, Asperger was “unobjectionable with respect to his character and politics.” His Catholic orientation was considered a minus, but this was mitigated by the fact that he had not been actively involved with the Christian Social Party or the Austrofascist regime. Crucially, the assessment concluded that Asperger “was in conformity with the National Socialist racial and sterilization laws” (Fig. 4).^39^

This investigation in all likelihood constituted the basis for Asperger’s claim, made 24 years later, that he had faced persecution by the Gestapo. Hamburger was certainly in a position to decisively influence the outcome of such a procedure, by vouching for his protégé’s willingness to cooperate with the regime—a less dramatic but much more plausible version than the alleged arrest, for which no documentary evidence exists. This explanation also correlates with Asperger’s 1974 account that Hamburger saved him “from the Gestapo” rather than “from being arrested by the Gestapo,” as he put it in 1962. If the latter story were true, it would be difficult to explain why Asperger (to the best of my knowledge) did not publicly mention it until 17 years after the war, although it would have benefitted both him and Hamburger.^40^ In all, this investigation is the only documented instance of political trouble for Asperger; the sources otherwise reflect a spotless record of political accommodation with National Socialism.

In this context, a crucial question concerns Asperger’s role in a truly heroic episode involving the pediatrician Josef Feldner (1887–1973), who over many years volunteered on the Heilpädagogik ward. In September 1942, he took in Hansi Busztin (1925–1996), a Jewish patient of his, and hid him until the end of the war. Unusually, Busztin lived a relatively open life, with regular visits to the public library and the opera; he estimated that around 100 people knew about him, many of whom provided support [51]. In a memoir written in the 1980s, Busztin referred to “a group of opponents of National Socialism” on the Heilpädagogik ward, “nearly all of whom knew” about him and “helped his later adoptive father in various situations.”^41^ Did Asperger belong to this circle of supporters? Busztin does not mention Asperger—and, interestingly, Asperger did not mention the episode even in instances where he was trying to establish his anti-Nazi credentials [3, 48] or in his 1975 obituary for Feldner [52]. Remarks published by Asperger in 1962 on the occasion of Feldner’s 75th birthday suggest, however, that he at least knew about Feldner’s activities, but did not play an active role in them:

It is clear that such a spirit had to be diametrically opposed to National Socialism. He acted accordingly. What he said and did during those years often made his friends’ hair stand on end. There are episodes—confrontations with the Gestapo, the hiding over years of a Jewish student whose family had been exterminated—which could have been taken from an adventure novel [53].

This episode could help explain why Asperger joined the military in March 1943.^42^ In the 1974 interview already mentioned, he claimed to have volunteered to escape reprisals from the Gestapo because he had refused to cooperate with Nazi race hygiene policies [3]. While this is contradicted by the favorable assessments he continued to receive from Nazi officials (for example during the vetting for his Habilitation), the cited evidence and the timeline of events suggest a direct connection—namely, that he wanted to get away from the Vienna clinic in case Busztin were discovered.

One of the main arguments for Asperger’s ostensible distance to National Socialism is the fact that he never joined the NSDAP.^43^ Given the high proportion of party members among non-Jewish physicians, this is certainly significant. This does not mean, however, that Asperger kept a principled distance from the NSDAP apparatus. In fact, he sought membership in several organizations affiliated with the NSDAP. According to a 1940 questionnaire, Asperger joined the *Deutsche Arbeitsfront* (German Labor Front, DAF) in April 1938, the *Nationalsozialistische Volkswohlfahrt* (National Socialist People’s Welfare Organization, NSV) in May 1938, and (as a candidate; see below) the *Nationalsozialistischer Deutscher Ärztebund* (National Socialist German Physicians’ League, NSDÄB) in June 1938. He also mentioned that he had committed himself to working for the Hitler Youth.^44^

The DAF and the NSV were mass organizations often used to demonstrate loyalty to the regime while avoiding the ideological commitment of NSDAP or SS membership. The NSDÄB was a different matter, however. It saw itself as the ideological spearhead of the Nazi Party within the medical profession, as an advisor to the NSDAP “in all questions regarding public health and race biology” and as a recruitment pool for medical positions in the party apparatus. While full membership was restricted to NSDAP members, other health professionals who supported the goals of the NSDÄB could obtain the status of candidates, as did Asperger [54].^45^

These memberships should be seen against the backdrop of the heavy Nazi influence at the clinic (see [4]: 120–1). Most likely, Asperger took these decisions in order to protect and further his career. By foregoing NSDAP membership, he chose a middle path between keeping his distance to the regime and outright alignment.

It is important to note that Asperger had all the political protection he needed through his mentor Franz Hamburger. Given the hierarchical structure of Austrian universities and the strong position of clinic chiefs, Hamburger was in a position to make or break Asperger’s career even under less complicated political circumstances. The political capital Asperger enjoyed thanks to Hamburger’s unwavering patronage was much stronger than anything he could have achieved on his own. Hamburger was one of the NSDAP’s figureheads within the Vienna medical school and had considerable clout within the Nazi medical establishment both in Vienna and—thanks to his position as president of the German Association of Pediatrics—in Germany generally ([4]: 129, 134). After the Anschluss, when the ban on the NSDAP was lifted, Hamburger could openly declare his allegiance to Adolf Hitler ([4]: 126). In a programmatic speech in 1939 (“National Socialism and Medicine,”) he revealed how central Nazi ideology was to his approach to medicine: “A teacher of obstetrics, a teacher of pediatrics, internal medicine, or neurology has to be a true National Socialist. He has to be completely permeated with the foundations of National Socialist life and health leadership” ([55]: 142). Asperger, without being a convinced National Socialist, clearly managed in Hamburger’s view to conform somehow to this highly ideological model of a physician.

As mentioned above, NSDAP functionaries on several occasions wrote confidential assessments of Asperger’s political orientation. Although they are the best sources available regarding Asperger’s attitude towards National Socialism and his standing in the eyes of the regime, these documents have not previously been examined. In all, they demonstrate how after an initial phase of distrust the party authorities came to see Asperger in an increasingly positive light. On 4 January 1939, for example, Asperger’s *Ortsgruppenleiter* (Local Party Group Leader) put the following on record: “no merits for the [Nazi] movement,” “attitude towards the NSDAP before the Anschluss indifferent,” “does not participate in public political life,” and “political orientation of the family Christian-Social”. It was noted positively that he had not taken any stance against the Nazi takeover in Austria. The *Kreisleiter* (District Party Leader) added to the same document: “his readiness to engage is only partially existent, because as a former Christian-Social he is quite indifferent.”^46^

Less than 2 years later, Asperger’s political evaluations had changed in tone, even if his past affiliation with the Christian-Social camp was still held against him. One of several similar documents from his NSDAP personnel file reads as follows:

In response to your enquiry from 25 October 1940 I declare that Dr. Asperger is a faithful Catholic, but without supporting the political tendencies of Catholicism. Although he was a member of the Catholic association ‘Neuland’, he had no common interests with the politicians of the [former Austrian] system. Regarding questions of the racial and sterilization laws he conforms to National Socialist ideas. With respect to his character and in political terms he is considered unobjectionable.^47^

Another high-ranking Nazi official’s evaluation from roughly the same time is similar in tone:

Dr. Asperger hails from Catholic circles and his orientation during the period of the [previous Austrian] system was strictly Catholic. He was a member of the Catholic organization ‘Neuland’ and of the physicians’ association ‘Lukas Guild’. He has never taken active steps of any kind against National Socialists, although it would have been easy for him to procure incriminating evidence at the Pediatric Clinic, which was staffed exclusively with Nazi physicians. In terms of his character, Dr. A. receives favorable descriptions.^48^

**Fig. 5:**
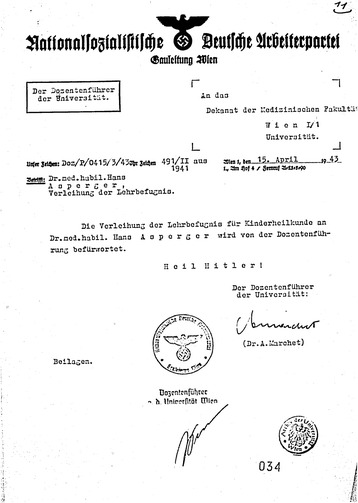
In April 1943, the Vienna chapter of the NSDAP’s *Nationalsozialistischer Deutscher Dozentenbund* (National Socialist German Lecturers’ League) approved Asperger’s application to receive his postdoctoral Habilitation. (UAW, MED PA 17, Asperger)

Due to his political past, the party hierarchy treated Asperger with a certain reservation. This changed over time, however, as he was increasingly regarded as politically reliable, and no obstacles to his career resulted. This development culminated in Asperger obtaining his Habilitation in 1943, the academic qualification necessary to become a lecturer (and, eventually, a professor). According to the Nazi doctrine, medicine should be based both on science and the ideology of National Socialism. Therefore, Asperger had to both submit a postdoctoral thesis (his work on “autistic psychopaths”) and pass political vetting by the *Nationalsozialistischer Deutscher Dozentenbund* (National Socialist German Lecturers League)—which raised no objections (Fig. 5).^49^ Additionally, since he had not obtained the title of *Facharzt* (medical specialist) in pediatrics, the NSDAP *Gauärzteführer* (Physicians’ Leader) of Vienna, Otto Planner-Plann (1893–1975), had to certify that he had the necessary qualifications. This is another indicator that Asperger enjoyed the trust of the highest ranks of the Nazi medical establishment in Vienna.^50^

After the Anschluss, Asperger tried to prove his loyalty to the new regime in various ways. In public lectures (which were later published), he argued in favor of his discipline’s mission within the Nazi state and declared his allegiance to the tenets of Nazi medicine (see the “‘The best service to our *Volk*’: Asperger and Nazi race hygiene” section). Indeed, Asperger went so far in these attempts that his collaborator, Josef Feldner, had to reign him in lest he risk his credibility: “Your lecture: the introduction is good as it is (maybe just a little bit too Nazi for your reputation). E.g., I would drop the thanks to the Führer. … I write what I have in mind, forcing myself to blow Hitler’s horn a little. Maybe you can make something of it.”^51^ Asperger’s case files also demonstrate how he tried to prove his loyalty. Beginning in 1938, he took to signing his diagnostic reports with “Heil Hitler”—a merely symbolic, but revealing gesture.^52^

### Asperger’s Jewish patients

The question of Jewish patients on Asperger’s ward has not been raised in the literature so far, despite the fact that their fate is relevant for a number of reasons. How they were diagnosed and what decisions about their future were taken at the clinic had an important impact on their chances of survival. The files on the Jewish children also provide insights regarding Asperger’s actions under National Socialism and on his general attitude towards Jews.

Jewish children were proportionately under-represented among the ward’s patients even before they were successively excluded from public medical institutions after 1938. Perhaps the strong Nazi influence that pervaded the clinic after Hamburger’s takeover in 1930 deterred Jewish parents from seeking the clinic’s services—although the case records of Jewish children from the decade before the Anschluss (16 in total), except for isolated instances of stereotyping, show no evidence of anti-Jewish bias.^53^

At the time of the Nazi takeover in Austria, two 13-year-old Jewish boys, Alfred S. and Walter Brucker, were patients at the ward. Alfred’s file contains no evidence that he was treated in any way differently from the other children. Admitted because of a scuffle at school, Asperger diagnosed the boy as an “autistic psychopath” on 22 March 1938. He found Alfred’s intellectual capabilities “above average in some respects” and recommended placing him with Jewish foster parents rather than returning him to his non-Jewish foster mother (whom Alfred liked). At the time, approximately 8000 children had been entrusted to foster families by Vienna’s youth welfare services. A number of these children—like Alfred—were Jews living with non-Jewish families. When the Nazis took over the city administration, this came to be regarded as a problem, and Jewish foster children were separated from their caretakers and segregated in Jewish orphanages, which for many became death traps during the Holocaust. Whatever his specific motivation might have been, in recommending Alfred’s placement specifically with Jewish foster parents, Asperger anticipated the Nazis’ official policy of segregation which took shape in the following years ([56]: 90, 101).^54^ At a time when Jews were subjected to humiliation and violence in the streets and anti-Semitism became official policy, the decision to highlight the boy’s Jewish background—for which no medical or pedagogical reasons were given—seems questionable. A safer alternative would have been to avoid any reference to Alfred’s biological family, although in retrospect, it is impossible to say whether this would have made a difference. The diagnostic report itself is rather benevolent in tone; Asperger considered Alfred capable of functioning among adults, who would feel less provoked by his behavior than children. Ultimately, Asperger’s recommendation was not followed, and Alfred was transferred to a Jewish orphanage. His fate is unknown.^55^

Walter Brucker was admitted to the clinic on 14 March 1938, the day following the Anschluss, because of extreme agitation. His record allows a rare insight into daily life on Asperger’s ward during these critical days. On 15 March, amidst cheering youngsters, Walter had to listen to a triumphant speech by Hitler. Despite the fact that as a Jew Walter had every reason to panic, his fearful reaction was held against him. The entry of that day (not in Asperger’s handwriting) stated that Walter “is much more disagreeable than three weeks ago, when he was [last] here. During Hitler’s speech, he put his head into his hands on the table and stared into a void. He was very agitated; when a child broke out in cheers he opened his eyes wide and turned pale.” Asperger’s diagnosis all but ignored the boy’s precarious situation and framed his mental troubles as follows: “severe psychopathy, with a particular sensitivity and paranoid irritability.” Asperger thus pathologized and de-politicized the boy’s reactions to the anti-Jewish persecution then pervading the city; based on the same logic, in a formulation that was perhaps meant as an act of generosity, he stressed that Walter could not be held fully responsible for his sometimes aggressive reactions. In his diagnosis, Asperger omitted the fact that Walter was Jewish and that his life was under threat from the Nazi regime. Although this is in line with Asperger’s general tendency to attribute mental troubles to “constitution” rather than environmental factors, in this particular case, it is possible that he was also trying not to highlight the boy’s Jewish background (in contrast to his actions in Alfred’s case). As it turns out, Walter indeed had every reason to be fearful. He died on 26 February 1945 as a slave laborer of “Projekt Riese,” the construction in Lower Silesia of underground facilities that included Hitler’s new headquarters [57].

As far as the written record is concerned, there is no indication that Asperger was guided by personal animosity towards Jews, but there is a notable absence of empathy for their plight under Nazi rule.^56^ The report he wrote in November 1940 on 11-year-old Ivo P. supports this interpretation. He stressed that the boy was “not constitutionally dissocial,” and that he had good potential, provided that he would be held under permanent supervision in an institutional setting. Almost as an afterthought, he added: “The only problem is that the boy is a *Mischling* of the first degree” (Nazi jargon meaning that he had one Jewish parent)—a piece of information that under the circumstances was extremely dangerous for the boy.^57^

Racial stereotyping became—not surprisingly—more frequent following the Anschluss. Marie Klein, admitted as a 9-year-old in late 1939, was described by one of Asperger’s assistants as a “normally developed, slightly underweight girl of Jewish appearance.” Asperger himself remarked that her manner of speaking stood “in contrast to her quite Jewish character” and noted on the cover sheet of her record that she was a “Mischling.” According to her file, Marie had never caused any trouble until she and her mother—who was a Catholic of Jewish descent—were forced out of their apartment in August 1938.^58^ They had to move to an asylum run by the Catholic relief organization “Caritas Socialis” for Catholics of Jewish descent and children classified as “non-Aryan.”^59^ From then on, Marie began to suffer from violent fits, which led to her admittance first to the psychiatric clinic and then to Asperger’s ward. When she spoke of the violent abuse she had suffered at the asylum, this was taken as an indication of her dishonesty rather than an explanation for the changes in her behavior.^60^ Two years after her transfer from the Heilpädagogik ward to a children’s home in February 1940, Marie Klein was deported to the Wlodawa ghetto, 11 km north of the Sobibor extermination camp. The precise time of her death is unknown, but in summer 1942, there was an “Aktion” specifically targeting Jewish children between 10 and 14 years of age (Marie would have been 12 by then), who were separated from their parents and killed in the gas chambers at Sobibor [58, 59].

Lizzy Hofbauer, a 12-year-old Jewish girl, was admitted in 1939 because of severe mental troubles: “Two days before admittance she acted as if insane, talked of anti-Jewish persecution, was in great fear, asked herself is she was confused or insane. She thought a Jewish acquaintance had died from hanging, but could be convinced that this was not true.” Asperger interpreted these signs of distress as symptoms of schizophrenia and wrote the following: “For her age and race conspicuously retarded sexual development.”^61^ These comments suggest that Asperger had at least partly internalized the sexualized anti-Jewish stereotypes circulating at the time.

This leads to the broader question whether Asperger held anti-Semitic views. Apart from the case files quoted above, there is scant direct evidence. On the one hand, hostility towards Jews and their alleged corrupting influence was a common ideological denominator of the groups Asperger associated with. Until the end of his life, as far as his public statements are concerned, he never distanced himself from the racialized anti-Semitism that pervaded Austrian and German political life during the twentieth century nor did he comment on the destruction this had brought down on the Jews of Europe during the Holocaust.^62^ On the other hand, Asperger worked closely with Jewish colleagues such as Anni Weiss and Georg Frankl before the Anschluss—a relationship that due to the tightly knit community at the Heilpädagogik ward went beyond the purely professional and was renewed after the war ([10]: 102–4, 109). Like many aspects of Asperger’s life, his relationship to Jews was fraught with ambivalence—and further complicated by the fact that his early career profited from the removal of so many Jewish colleagues, including those he called his friends.

### “The best service to our *Volk*”: Asperger and Nazi race hygiene

Although Asperger published at least a dozen papers during the Nazi period, the existing (especially English-language) literature focuses almost exclusively on two of these: “The Mentally Abnormal Child” from 1938 and “The Autistic Psychopaths in Childhood” from 1944 [1, 2]. In the following, I will broaden this narrow scope and present an analysis based on the full range of Asperger’s published statements on politics, race hygiene, and the role of Heilpädagogik in society. I will show that Asperger on several occasions supported tenets of Nazi race hygiene and medicine, contributing to their legitimization.

Among Asperger’s Nazi-era publications, the 1938 paper stands out for several reasons. Published 5 years before Leo Kanner’s famous 1943 article on autism, it contains the first account in the scientific literature of “autistic psychopathy” as a not previously described syndrome. As the written version of a lecture held less than 7 months after the Anschluss, it also reveals how Asperger positioned himself vis-à-vis the new rulers as someone who could be trusted to adapt to the new political situation. Crucially, Asperger opened with an endorsement of National Socialism’s anti-individualistic and totalitarian approach to medicine and health:

We stand in the midst of a massive reorganization of our intellectual and spiritual life, which has seized all areas of this life—not least in medicine. The central idea of the new Reich—that the whole is more than its parts, and that the *Volk* is more important than the individual—had to bring about fundamental changes in our whole attitude, since this regards the nation’s most precious asset, its health ([1]:1314).

Before the initiation of “euthanasia” killings in 1939, the most serious consequence of these ideas was the Law for the Prevention of Hereditarily Diseased Offspring of July 1933, under which 220,000 individuals had already been forcibly sterilized in Germany by the beginning of 1938 ([60]: 233).^63^ Asperger’s audience of physicians were well aware of these policies, which had been widely debated in medical circles, so they must have understood what he meant by the following exhortation to cooperate with the regime’s sterilization policies:

You know by what means one strives to prevent the transmission of diseased hereditary material—many cases that belong here are hereditary disorders—and to promote hereditary health. We physicians have to take on the tasks that accrue to us in this area with full responsibility ([1]:1314).

Asperger repeated this motive of “responsible cooperation” with Nazi race hygiene in later writings. We will see shortly what these tasks entailed and how he handled them in the context of his own work. In his lecture, he went on to specify how the sterilization law should be implemented with regard to those children who had “features opposed in nearly every way” to the high-functioning autistic type described for the first time in the paper:

These children are intellectually below average (including to the degree of feeble-mindedness)—whereby with intelligence we mean abstract intelligence—whereas practical reason, in short everything that has to do with instinct, including the practical usefulness and the values of character, are much better developed in relative terms. These cases are important—or at least they will be as soon as the Law for the Prevention of Hereditarily Diseased Offspring comes into force here. If the physician has to take a decision in such a case, he will not be allowed to do so based solely on a questionnaire or the intelligence quotient. Rather, he will base it primarily on his knowledge of the child’s personality, taking into account all of the child’s skills in addition to abstract intelligence ([1]:1317).

This passage has been quoted as evidence that Asperger tried to publicly protect his patients from forced sterilizations ([8]: 364; more cautiously [9]: 206–7). This call for restraint is all the more significant given the pending investigation into Asperger’s political reliability (closed in June 1939, see above). Why did these comments then not hurt his standing in the eyes of the Nazi hierarchy, which came to the conclusion that he was in conformity with Nazi race hygiene policies?

It should be noted that calling for a “holistic” approach to children’s personalities was not unusual as such—it was in fact characteristic of the approach at the Heilpädagogik ward since Lazar’s days. In the ideological context of post-Anschluss Vienna, putting *Gemüt* (“soul” or “character”) and “practical intelligence” over “abstract intelligence,” far from being out of line, corresponded with the Nazis’ overall disdain for analytical thought, which they connoted as “Jewish.” Indeed, the official legal commentary on the sterilization law defined “feeble-mindedness” along similar lines ([61]: 119). The notion of *Lebensbewährung* (“probation in life”), which the sterilization courts applied in cases with unclear heredity, also underlines how race hygiene policy was guided by a “holistic” approach to intelligence ([62]: 124). In 1940, practical skills and “performance” became the decisive criteria in decisions on race hygiene measures.^64^

It is important to note that Asperger focused on skills, where others were primarily concerned with defects. Overall, however, assessing “hereditary value” according to a range of criteria rather than intelligence alone could cut both ways for the patients; those classified as “autistic psychopaths” may well have scored better on intelligence alone. While Asperger’s 1938 speech should not be misconstrued as a fundamental critique of race hygiene, it is an example of how he managed to formulate certain concerns without violating the boundaries of the politically acceptable.

Another of Asperger’s publications (from 1939) captured in a nutshell the central tenets of Nazi medicine, including its typically euphemistic language, as in “restrictive measures”:

In the new Germany, we physicians have assumed an abundance of new responsibilities in addition to our old ones. To the task of helping the individual patient is added the great obligation to promote the health of the *Volk*, which is more than the welfare of the individual. I do not need to elaborate on the enormous dedicated work being performed in terms of positive, supporting measures. But we all know that we also have to carry out restrictive measures. Just as the physician often has to make painful incisions during the treatment of individuals, we also have to perform incisions on the national body [*Volkskörper*], out of a sense of great responsibility: We must ensure that the diseased who would transmit their diseases to remote generations, to the detriment of the individual and of the *Volk*, are stopped from transmitting their diseased hereditary material ([63]:943).

The potential impact of the Nazi race hygiene paradigm on Asperger’s work was to a large extent determined by the role inheritance was thought to play in the transmission of personality traits and mental disorders. In this regard, Asperger stressed the benefits of optimal environmental conditions (such as those present at his clinic) even when hereditary makeup (what he referred to as “constitution”) was defective:

Therefore we are called more than others to contribute decisively to what is probably the most important area of research on human heredity, namely the questions concerning the inheritance of mental traits and mental abnormalities. We must also lead the way in the practical tasks of eugenics, especially with regards to the problems relating to the Law for the Prevention of Hereditarily Diseased Offspring—and not just the physicians, but also the special school teachers we work with. But we also have […] opportunities few others have to study the decisive question: ‘What influence do optimal environmental conditions have on hereditarily burdened individuals, what can “education in spite of inheritance” achieve, is pedagogic work with individuals outside the norm worth the trouble?’ ([49]:353)

Although many race hygienists were more dogmatic in terms of a one-sided genetic determinism, Nazi ideology was not monolithic. Asperger’s flexible approach was not only compatible with hardline measures such as forced sterilizations (as this passage illustrates), it was also in line with other powerful currents such as the Hitler Youth’s paradigm of pedagogy and leadership or the mainstream of Nazified Heilpädagogik ([64]: 161–6, 178–92).^65^

In his 1944 paper on autism, Asperger reiterated his belief that an individual’s possibilities of mental development were primarily determined by their genetic makeup; thus, Heilpädagogik could only hope to achieve improvements within these predetermined parameters: “It has been firmly established that psychopathological states are anchored in the human constitution and are therefore inheritable; yet it has also been established that it is vain to hope to find a clear, simple mechanism of inheritance” ([2]: 135). While this view has stood the test of time with regard to specific disorders such as autism, it was seriously misguided in other cases, for example, when Asperger diagnosed children as young as five in terms reminiscent of Lambroso’s “born prostitutes” or “born criminals.” In cases of sexual abuse, he often tended to blame the victims, based on the notion of constitutionally determined patterns of behavior which supposedly encouraged (or “seduced”) the perpetrators.^66^

A key element in the established narrative of Asperger as a principled opponent of Nazi policies derives from his repeated appeals to treat troubled children with the utmost dedication to help them overcome their challenges ([20]: 17, [21]: 127–9). A number of Asperger’s publications indeed convey an attitude of sympathy towards his patients, and on several occasions, he pleaded for tolerance and attention towards them. One of the most significant passages in this regard is contained in his 1944 paper on autism:

We think that such individuals have their own place in the organism of the social community, which they fully occupy, some of them maybe in ways nobody else could. […] Such individuals show more than others what capacities for development and adaptation even abnormal personalities dispose of. Often, in the course of development, possibilities for social integration arise which one would not have expected before. […] This fact determines our attitude and our value judgment towards difficult individuals of this and other kinds and gives us the right and the obligation to stand up for them with the whole force of our personality ([2]:135).

This is in line with his 1938 speech, in which he also expressed his determination to side with his patients:

But let me discuss this problem today not from the standpoint of the *Volk* as a totality—in this case one would have to primarily focus on the Law for the Prevention of Hereditarily Diseased Offspring—but from the standpoint of the abnormal children. How much can we accomplish for these children shall be the question ([1]:1314).

Again the question is whether this approach put Asperger at odds with the regime or even made him vulnerable to reprisals, which is a central claim in the narrative of his resistance to the Nazis. The evidence, however, does not support this. Indeed, the fact alone that Asperger’s statements continued to be published in journals controlled by Nazi loyalists shows that they were not perceived as critical of the regime. Furthermore, Asperger’s career advanced unhindered during this period. Repeatedly vetted for promotion, he received positive assessments regarding his political reliability, as discussed in the “Political trajectory after the Anschluss in 1938” section.

Most importantly, it is a misunderstanding that therapeutic support for “abnormal” children had no place in the Nazi state, determined as it was to exterminate mentally disabled individuals. Due to increasing labor shortages, it became a political and military imperative to rehabilitate as many potential workers as possible, even those considered of inferior hereditary quality. In the context of “euthanasia,” the extermination of “incurable” patients—after attempts to improve their condition had failed—coincided with an increased interest in “active therapy.” The dichotomy of murder and therapy is exemplified in the introduction of electroconvulsive therapy, which was promoted by “T4,” the organization responsible for the gassing of tens of thousands of patients, to reduce the residual group of “incurable” patients [65].^67^ In this light, Asperger’s pleas to spare no effort in educating and guiding “difficult” children were not necessarily a challenge to Nazi pedagogy and race hygiene; rather, this was easily compatible with the Nazi state’s aim to control, discipline, and organize children and youths deemed “worthy” of belonging to the *Volksgemeinschaft* (“people’s community”). This was stressed by Asperger himself, who repeatedly insisted on the productive role Heilpädagogik could play within the new Nazi order, including in his 1938 speech:

And if we help them [the abnormal children] with all our dedication, we also render the best service to our *Volk*; not only by avoiding that they burden the *Volksgemeinschaft* with their dissocial and criminal acts, but also by trying to ensure that they fulfill their duties as productive individuals in the living organism of the *Volk* ([1]:1314).

Indeed, even the most virulent Nazis among Asperger’s colleagues endorsed therapy for those seen as potential assets to the state. This includes Asperger’s mentor Franz Hamburger and also applies to Erwin Jekelius, a pediatrician trained at Hamburger’s clinic, who in 1940 became the main organizer of the “T4” killing operation in Vienna. He made sure that local authorities and hospitals cooperated and that the operation ran smoothly. From June 1940 to the end of 1941, Jekelius directed the child killing facility Am Spiegelgrund, where hundreds of disabled children were murdered.^68^

Jekelius had received part of his training at the Therapeutic Pedagogy Ward under Asperger’s direction, where he was employed from August 1933 to February 1936 ([10]: 102, 49). The two men maintained professional contacts during the Nazi period. In 1941, when Jekelius became the first chairman of the newly established Viennese Association for Therapeutic Pedagogy, Asperger represented the Pediatric Clinic along with Hamburger ([4]: 172–3). At Vienna’s Main Health Office, where Jekelius directed a unit in charge of “the mentally ill, psychopaths, and addicts”—a position that he used as a cover for his activities for “T4”—Asperger on 1 October 1940 began to work (part-time) as a medical specialist and evaluator of children with mental “irregularities” (*Auffälligkeiten*). One document in Asperger’s personnel file suggests that in this capacity, he was attached to Jekelius’ unit, while others place him in a different unit of the same department, a discrepancy possibly due to disruptions in the city administration during this period.^69^ In any case, the fact that Asperger was named as a member of Jekelius’ staff suggests that he obtained the position on Jekelius’ recommendation or at least with his consent. Due to a lack of sources, the exact nature of Asperger’s work for the city in this context (and of his collaboration with Jekelius) remains unclear—with the crucial exception of the screening of more than 200 children in a mental institution in Gugging near Vienna, many of whom were sent to die at Jekelius’ Spiegelgrund institution (see the “Limits of ‘educability’: Asperger and the Spiegelgrund ‘euthanasia’ facility” section).

Erwin Jekelius represents Nazi medicine in its most inhumane extremes: a fanatical Nazi and a murderer responsible for the deaths of thousands of patients. Had Asperger deviated from the party line, Jekelius would certainly have reined him in. Instead, this is what Jekelius had to say about Asperger and his therapeutic approach:

At this opportunity, I would like to remind you of the substantial lecture on therapeutic pedagogy which our Dr. Asperger gave last year in this same place. He explained in a vivid and convincing way that especially in the Third Reich, with an abundance of new tasks and a lack of manpower, one cannot relinquish those who ‘stand at the margin’. He mentioned impressive examples of former patients at the Heilpädagogik ward who proved themselves brilliantly on the internal and external front during the great struggle for the final liberation of our German people. And more than one former ‘problem child’ who today bears the Iron Cross for valiant behavior before the enemy would probably have gone to the dogs had one not taught him according to therapeutic pedagogic principles how to defeat the inner enemy ([66]:386).

To be sure, when their writings are set side by side, there is an enormous divide between the two men in tone. This is also apparent in the following passage, in which Jekelius states what should be done with children not deemed treatable with Heilpädagogik: “The idiot is sent to an asylum, and the anti-social to a concentration camp for minors” ([66]: 385).^70^ This is far harsher language than Asperger ever used, who instead emphasized empathy for “abnormal” children. But as Jekelius’ approving nod to Asperger indicates, even he agreed on the role of Heilpädagogik in rehabilitating troubled children in order to turn them into productive members of the German body politic.

This utilitarian approach, broadly accepted as the raison d’être of Heilpädagogik, is a leitmotif throughout Asperger’s writings during the Nazi period and beyond:

I wanted to emphasize this from the beginning, when I talk today about our Aesculapian obligation specifically towards the psychologically abnormal individual, as I see this obligation. […] The question is: is the task of caring for intellectually or personally abnormal individuals worth our full commitment? […] The mentioned facts show us well enough that we often have to be very careful with the disdainful verdict of ‘inferior value’ and potential consequences that might follow from it. […] But if we care for these people—and be it with painful commitment and willing to make sacrifices—we will be able to take at least part of them to a point where they will not be a burden and danger to the national community, but its productive members ([63]:944).

In the 1941 paper referred to by Jekelius, Asperger defined the relationship between his own professional credo and the Nazi state’s pedagogic program in even more explicit terms:

Our time has brought revolutionary changes in the field of education: Whereas in earlier times a number of philosophical, political, and religious orientations stipulated their pedagogic goals and consequently were in competition with each other, today National Socialism has established its pedagogic goal and demands that it be the only valid one. As much as this development is to be approved, we nevertheless have to stress: This one goal, the integration into the National Socialist state, can only be achieved with these children by using different means. […] From innumerable reports and visits, also from letters from the front, from soldiers’ visits we know how many of our former children, including very difficult cases, entirely fulfill their duties in their professions, in the armed forces, and in the [Nazi] party, not a few among them in eminent positions. This is how we know that the success of our work is worth the effort [67].^71^

This paper was originally presented in September 1940 at a prominent pediatric conference in Vienna. Asperger was one of only three speakers from Vienna. The keynote speaker was Reich Health Leader Leonardo Conti (1900–1945).^72^ While this helps explain Asperger’s references to the war effort and the party, it also demonstrates that he was deemed trustworthy enough to represent his field in such a prominent forum and that the positions he adopted were by no means considered unacceptable or even controversial by the Nazi hierarchy. On the same occasion, Werner Villinger (1887–1961), the founding father of youth psychiatry in Nazi Germany and an expert evaluator for the “T4” killing campaign, expressed the dichotomy between “education” and “elimination”: “Only where this [successful educational attempts] proves to be impossible, a weeding out needs to take place, with permanent internment in a work colony of sorts” ([68]: 1161).^73^ The complex and sometimes contradictory attitude towards children with disabilities or other challenges is underlined by the fact that the Hitler Youth had special formations for the blind and the deaf ([64]: 166–75). Overall, as we have seen, it was not in dispute that Heilpädagogik had an important role to play in helping alleviate the acute shortage of manpower that threatened Nazi Germany’s war effort.

The decisive question that remained was what should happen to the residual group of children whose disabilities were so impairing that efforts towards rehabilitation could not be justified under the utilitarian approach dominant at the time, and also professed by Asperger. While Jekelius explicitly mentioned “concentration camps” (for the rebellious) and “asylums” as a last resort (omitting the extermination of disabled children then taking place under his direction), Asperger chose to remain silent on this matter. This has critical implications, not least with regard to the relatively small subset of his patients whom he labeled as “autistic psychopaths.”^74^ Some authors argue that Asperger put the spotlight on children at what is often called the “high-functioning” end of the spectrum, interpreting this as a tactic to protect all children with autistic traits from race hygiene measures (e.g., [21]: 129, 216). This argument is problematic for several reasons.

First, the idea that Asperger tried to protect autistic children from Nazi race hygiene cannot be easily reconciled with the fact that he dedicated a section of his 1944 paper to the hereditary basis of the condition, insisting that “any explanation based on exogenous factors is absurd”. While this position anticipated later advances in autism research, the question arises whether under the circumstances it was prudent to put such an emphasis on heredity. Had protecting his autistic patients been his primary goal, he could have taken a more flexible position, one less likely to draw the attention of race hygienists to his patients ([2]: 128–32).

Second, his prognoses for the “autistic psychopaths” were far from universally optimistic. In his 1938 paper “The Mentally Abnormal Child,” he presented two boys. One boy was “intelligent far beyond his age” but suffered from mental and physical “oversensitivities” (no link to autism). The other represented the first case of an “autistic psychopath” in medical literature. Like the first boy, he exhibited “a contrast between pathological and in some ways valuable traits,” but as Asperger insisted, he suffered from a “profound disorder of the personality.” In this article, Asperger did not highlight the potential of “autistic psychopaths” but rather contrasted them unfavorably with other, less impaired patients. Even if the boy Asperger chose as an example of “autistic psychopathy” clearly belonged to the “high-functioning” group, Asperger emphasized that the condition varied greatly in terms of “social prognosis” and “worthiness.” While he deemed some of the “autistic psychopaths” capable of “great intellectual achievements,” in other cases, “autistic originality” was deemed “bizarre, eccentric, and useless”, with “fluid transitions towards schizophrenia” whose “main characteristic is also autism, the loss of any contact with the surroundings” [1]. The four boys featuring in his better known 1944 paper also varied considerably regarding the degree of their impairments, belying the notion that he focused on the most promising cases in order to present “autistic psychopathy” in a mostly favorable light. Fritz had (for his age) outstanding abilities in mathematics, but was incapable of attending regular school, having passed the first three school years by homeschooling. Harro’s autistic symptoms were less severe, but despite his good intellectual potential, he also had great difficulties concentrating and learning in the traditional school setting. The best hope for “autistic psychopaths,” according to Asperger, was to find a way to compensate for the lack of “instinctive social adaptation” via the intellect. The problem was, however, that the “autistic character” also occurred in the “intellectually lesser able, and even in the severely feeble-minded” ([2]: 85–103). In the case of Ernst, Asperger expressed “doubt whether he was particularly smart or feeble-minded” (he struggled to keep up even in special school). But, as Asperger insisted, there were “numerous clearly feeble-minded children who also exhibited the unmistakable features of the autistic psychopath” ([2]: 108). These latter cases, according to Asperger, were often very similar to conditions caused by organic brain damage such as birth trauma. He illustrated this with the fourth case in his study, Hellmuth, whom he described not as an “autistic psychopath” but as an “autistic automaton” ([2]: 110–1).

One could argue that even though Asperger mentioned children with impairments so severe as to exclude them from any useful place in society, he nevertheless embellished the overall picture of “autistic psychopathy” as far as his scholarly standards would allow him. Indeed, he insisted that only a small number of “autistic psychopaths,” those additionally burdened with a “clear mental inferiority,” were incapable of at least some degree of social integration. Nevertheless, the argument that Asperger focused on the better-functioning cases in order to protect all of his patients (presumably, by deflecting attention from the less well-functioning) is questionable given that Asperger by no means withheld from his readers the severe impairments of some of the boys.

Third, there is a fundamental flaw in the assumption that highlighting the potential of some of his patients would benefit all of them. The children at the lower end of the spectrum did not benefit from the potential ascribed to those on the higher end, even if they shared the overall diagnosis of “autistic psychopathy.” Their fate did not depend on the diagnostic label but on the individual assessment of their skills or disabilities. If anything, the utilitarian argument of “social worth” employed by Asperger (and by many of his colleagues) increased the danger to those children who could not fulfill these expectations. Focusing on the higher functioning children did nothing to lift the boat for all of them; those on the lower end still risked being left to drown. Often, Heilpädagogik’s function in this context was to decide where to draw the line.

The preference for children who could be expected to respond positively to pedagogical intervention and the exclusion of the “hopeless” was a feature of Heilpädagogik from its inception in early twentieth-century Austria. It is important to keep in mind that the mission of Asperger’s Heilpädagogik ward was primarily to deal with “difficult” children who caused problems that their caregivers were unable to confront without professional help.^75^ Children with severe mental disabilities were considered to fall outside the remit of Heilpädagogik since they promised no tangible progress. At the 1935 founding meeting of the Austrian Society of Heilpädagogik, Theodor Heller (1869–1938), one of its most influential figures, stated: “Curative pedagogy, however, will only attend to the educable elements and must hardly burden itself with the care for idiots.” The “uneducable” should be cared for in special institutions on humanitarian grounds, by contrast to the rationally and economically justified efforts of Heilpädagogik for the “educable” ([69]: 8–9).^76^ During the Nazi period, *Bildungsunfähigkeit* (uneducability) became the key criterion in the child “euthanasia” program [70].

Highlighting the potential of some patients should not then be mistaken for the defense of all children with disabilities. Rather, it served to point out the usefulness of Heilpädagogik to society. Also, Asperger did not adopt this strategy in reaction to the Nazi takeover in Austria. A 1937 paper (with the same title he would use in 1938) already used similar arguments in support of Heilpädagogik’s mission [71]. It is not hard to see why in the post-Anschluss climate Asperger felt it necessary to explain what he and his discipline had to offer to the new political regime, by stressing his allegiance to the fundamental principles of National Socialism and by adapting earlier arguments of Heilpädagogik’s utilitarian mission to the new political realities.

Overall, Asperger’s pleas to devote the best possible care to “abnormal” children did not place him outside the mainstream of Heilpädagogik and the nascent discipline of youth psychiatry under National Socialism. The papers presented to the first conference of the newly founded German Society for Child Psychiatry and Heilpädagogik, held in Vienna in September 1940, also reveal that Asperger’s positions clearly aligned with the opinions considered legitimate at such a representative forum. Although some of the speakers emphasized Heilpädagogik’s role in implementing mechanisms of race hygienic selection, the positive aspects of helping children reach their potential (within the limits set by their inherited constitution) also had a prominent place. If Asperger’s writings on his patients stood out because of their warmer tone, nothing, however, of what he said was out of line with the officially sanctioned discourse ([72]; for details, see also [73]).^77^

The adaptation of the Viennese brand of Heilpädagogik to the new political order and its race hygiene paradigm was facilitated by the fact that, since 1930, Hamburger had purged the influence of currents such as psychoanalysis and established the predominance of a purely biologistic paradigm based on the importance of inherited “constitutional” defects [74, 75]. Asperger, who had begun his career under Hamburger, shared many of these views, including a staunch opposition to psychoanalysis [76].

Heilpädagogik, then, was not only compatible with the Nazi state’s goal of building a national community (while excluding “unworthy” and “racially alien” elements); there was even an increased demand for experts willing to draw the line between those who could be expected to become useful members of this community and those who should be cast aside ([73]: 184). This increased demand, together with the exclusion of Jewish doctors,^78^ led to additional career opportunities for Asperger, for example, his appointment as an expert witness in May 1938 to the Vienna Juvenile Court.^79^ As mentioned, in October 1940, he additionally acquired a part-time position at the Public Health Office as the city’s medical specialist for “abnormal children,” a function related to Vienna’s special school system.^80^ In this capacity, he routinely wrote expert opinions which are hard to reconcile with his 1974 claim not to have reported patients to this office [3]. According to Hamburger, Asperger’s expert opinions were regarded as the “highest authority” not only by the youth welfare office and the youth court but also by the National Socialist Welfare Organization (NSV).^81^

Public health offices in Nazi Germany systematically collected information for a “hereditary index” (*Erbkartei*) of the whole population, designed to direct race hygiene measures against those deemed of inferior hereditary quality.^82^ Staff at the Spiegelgrund “euthanasia” facility routinely reported patients in this context.^83^ The records from Asperger’s ward, by contrast, contain only a small number of such documents.^84^ This would indicate that Asperger was indeed reluctant to report his patients for the “hereditary index”—provided that the records have not been purged of incriminating documents, which cannot be ruled out, since they were kept at the clinic where Asperger was director from 1946 to 1949 and again from 1962 until his retirement in 1977 [77]. In some cases, however, he demonstrably cooperated in such matters. At least seven patient files from his ward contain correspondence with the Public Health Office’s Department for Hereditary and Racial Care (*Erb- und Rassenpflege*), in four cases signed by Asperger personally. There is no indication as to why the department responsible for the “hereditary index” was involved in these cases but not in so many other similar ones.^85^

A sample of 30 patients admitted both to Asperger’s ward and to Spiegelgrund allows for a comparison of how children were diagnosed at the two institutions. In the next section, we will see what this comparison reveals about Asperger’s diagnostic approach, not least with regard to his often-claimed “pedagogical optimism” (details on the sample are also offered there). Regarding references to heredity and race hygiene, the result is the following: In 14 of the 30 cases, the files contain reports suggesting a hereditary factor in the child’s condition. In two of these cases, both Asperger and the Spiegelgrund staff suggested a hereditary etiology. In two other cases, Asperger included a reference to heredity in his report, but the Spiegelgrund staff did not. In ten of the cases, however, only the Spiegelgrund doctors referred to heredity. In the four cases in which Asperger referred to a hereditary factor, he did so by using the term “degenerative” (for example ascribing to the children a “degenerative constitution” or “degenerative personality”), without however suggesting any eugenic measures such as sterilization.^86^ The doctors at Spiegelgrund were clearly more inclined to refer to heredity, either directly as an alleged etiological factor or indirectly by including negative information on the child’s *Sippe* (kin).

Neither the Spiegelgrund files nor the case records from Asperger’s own ward contain evidence that he ever reported one of his patients to the Public Health Office for the purpose of sterilization.^87^

These findings support Asperger’s claim of non-cooperation with the sterilization program, though here, too, we must allow for the possibility of files having been purged. This raises the question whether this non-cooperation by omission should be considered a form of resistance. It is important to note that the sterilization program in Nazi Austria was never implemented on a scale comparable to Nazi Germany between 1934 and 1939 and that children were not its primary targets. In Vienna, the Hereditary Health Court decreed a total of 1515 sterilization cases. Although in one case a child as young as 13 was ordered to be sterilized, 83% of victims were older than 20 ([78]: 97, 144).^88^ Failure to comply with the sterilization law was widespread at the time, and there are no indications that this carried personal risks such as persecution by the Gestapo ([78]: 115). In 1942, the Public Health Office complained to the director of the general hospital (to which Hamburger’s pediatric clinic belonged) that the hospital’s clinics often failed to report patients with hereditary conditions ([78]: 116). Also, for those of Asperger’s patients who were admitted to the clinic, this responsibility fell on Hamburger as the director, shielding Asperger from—in any event unlikely—consequences.^89^

Crucially, only one of the surviving patient records from Asperger’s ward contains an explicit reference to the sterilization law; the documents are in accordance with Asperger’s publicly stated position on sterilization, calling for “responsible implementation.” In 1940, Asperger wrote a diagnostic opinion on Therese B., a 16-year-old former patient whose father wanted to have her sterilized because of alleged promiscuity. Asperger diagnosed her with psychopathy and “traits of nymphomania,” but pointed out that strictly speaking, she did not fall within the purview of the sterilization law, since her behavior had in all likelihood been caused by an earlier encephalitis, and not by a hereditary defect.^90^

More problematic is a report on a 15-year-old deaf-mute boy which Asperger addressed to the Health Office’s Department of Hereditary and Racial Care in March 1942. Under the category “kin,” Asperger listed various cases of deaf-muteness among Ernst’s relatives.^91^ Although Asperger did not explicitly refer to sterilization, the information provided meant that the addressee would have to initiate a sterilization procedure on the grounds that the condition appeared to be hereditary.^92^ Asperger could have omitted this information without any risk, but in this case (as in those he referred to Spiegelgrund, analyzed in the next section), it seems that he was willing to cooperate as long as he did not have to take direct responsibility for the consequences.

There is one case in which documents suggest that Asperger may have helped shield a patient from possible persecution. In the fall of 1939, he examined Aurel I., the 14-year-old son of a civil servant, who showed “behavioral peculiarities.” In his report, Asperger wrote that the boy would suffer mental and physical damage if placed among a group of children, which resulted in his exemption from school. His family then moved him to the countryside, where he spent the war in the care of relatives. In a 1962 letter, his sister credited Asperger with having saved Aurel from “castration” and possibly worse.^93^ Asperger wrote his report just days before the sterilization law was introduced in Austria, an event that was widely publicized [79]. In 2009, a Cologne art dealer (who had bought Aurel’s estate of drawings and papers) wrote to Asperger’s daughter speculating that Aurel, who after the war was diagnosed with schizophrenia, might have shown autistic traits when examined by Asperger.^94^ Ultimately, it is impossible to say with certainty what happened in 1939, and to what extent the dramatic elements of the story are a product of the years that passed before the quoted letters were written.

What emerges from the available sources is that Asperger’s approach to the forced sterilization program was ambivalent. On the one hand, as mentioned, he publicly signaled his fundamental agreement with the policy while calling for its cautious and “responsible” implementation—which is consistent with his overall strategy to demonstrate his willingness to cooperate with the regime without taking hardline positions on race hygiene. At the same time, based on his ward’s patient records, it appears that he abstained from reporting children for sterilization—a stance that does not seem to have put him at odds with the Nazi authorities, given the circumscribed implementation of the sterilization law in Austria. By the time the first procedures under the sterilization law were carried out in Austria in the fall of 1940, the prevention of “hereditarily diseased offspring” could rely on another, more radical method; with the establishment of Am Spiegelgrund in July 1940, the child “euthanasia” program had a dedicated killing facility in Vienna. Even if the vast majority of Asperger’s patients did not suffer from the degree of mental disability that the program was designed to eradicate, a number of them were killed at Spiegelgrund. His role in this context is the topic of the next section.

### Limits of “educability”: Asperger and the Spiegelgrund “euthanasia” facility

In his wartime publications, Asperger appears as someone who declared his willingness to cooperate with the Nazi state, propagated—albeit less enthusiastically than others—elements of Nazi race hygiene, and tried to argue that his discipline had an important role to play within the new political order. His main argument was the ability of Heilpädagogik to turn troubled, difficult, or “abnormal” children into useful members of society. His professed pedagogical optimism reached its limits, however, in children with greater degrees of mental disability. Although in his ward he usually dealt with more promising cases, in the course of his manifold activities as an expert for “abnormal” children, he was also confronted with children for whom the Nazi state had little more in mind than discreet medicalized extermination. In this regard, as we will see, his record was mixed.

Am Spiegelgrund was founded in July 1940 on the premises of Vienna’s Steinhof psychiatric hospital, after approximately 3200 patients had already been sent to the T4 killing facility in Hartheim [80]. The new facility was headed by Erwin Jekelius, Asperger’s former colleague at the university clinic. During Jekelius’ tenure at Spiegelgrund, the facility became a collecting point for children who did not conform to the regime’s criteria of “hereditary worthiness” and “racial purity.” From 1940 to 1945, nearly 800 children perished at the institution; many of them murdered by poisoning and other methods [81].

**Fig. 6:**
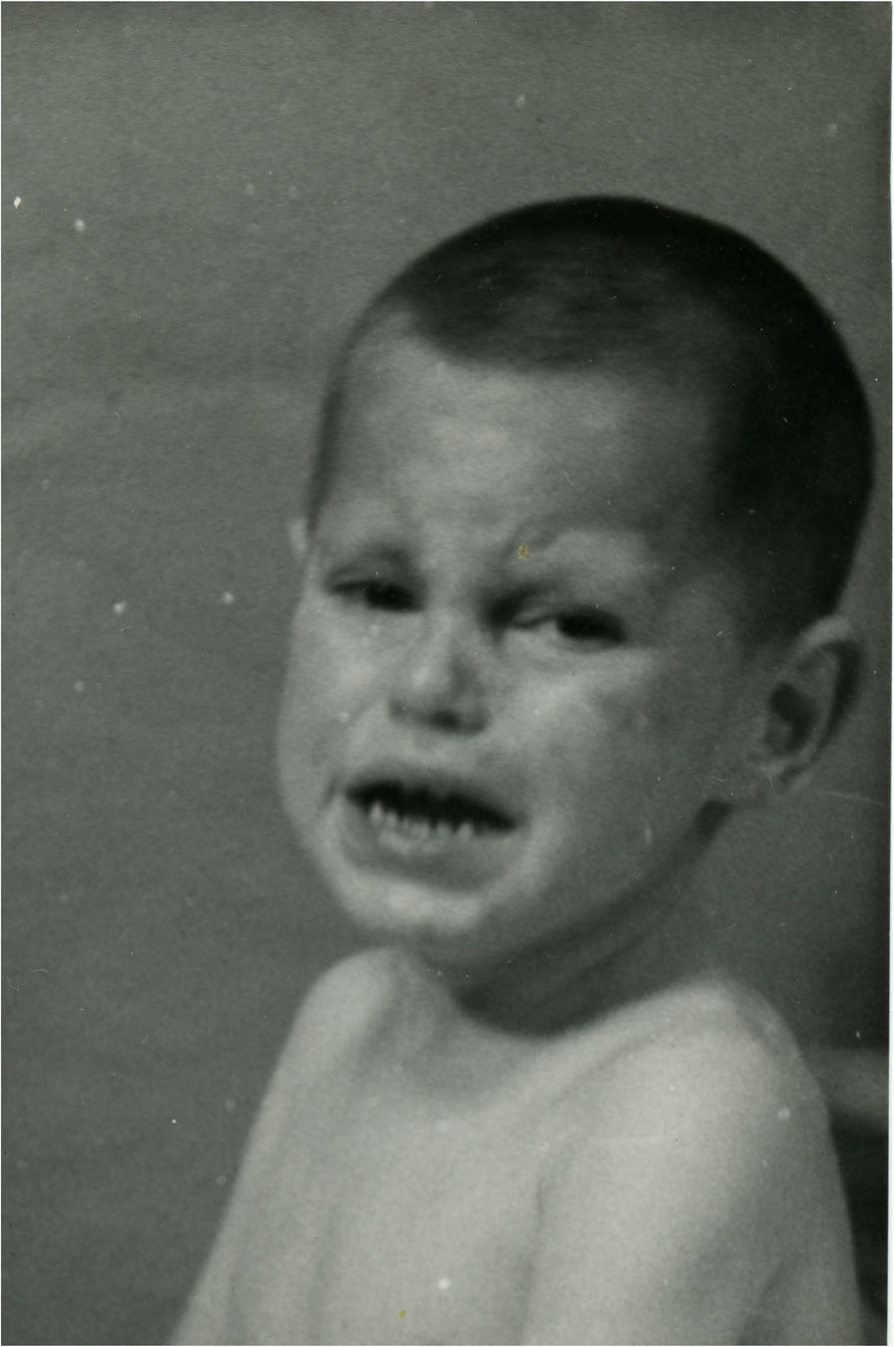
Herta Schreiber at the Spiegelgrund “euthanasia” clinic, where she died 3 months after admission (photo has been cropped) (WStLA, 1.3.2.209.10, Nervenklinik für Kinder, Krankengeschichte Herta Schreiber)

**Fig. 7:**
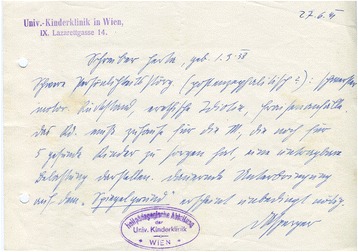
Hans Asperger recommended Herta’s transferal to Spiegelgrund because she “must be an unbearable burden to her mother,” 27 June 1941 (WStLA)

On 27 June 1941, 2 months before her third birthday, Asperger examined a girl at his clinic named Herta Schreiber (Fig. 6). The youngest of nine children, Herta showed signs of disturbed mental and physical development ever since she had fallen ill with encephalitis a few months before. Asperger’s diagnostic report on Herta reads as follows:

Severe personality disorder (post-encephalitic?): most severe motoric retardation; erethic idiocy; seizures. At home the child must be an unbearable burden to the mother, who has to care for five healthy children. Permanent placement at Spiegelgrund seems absolutely necessary.^95^ (Fig. 7)

**Fig. 8:**
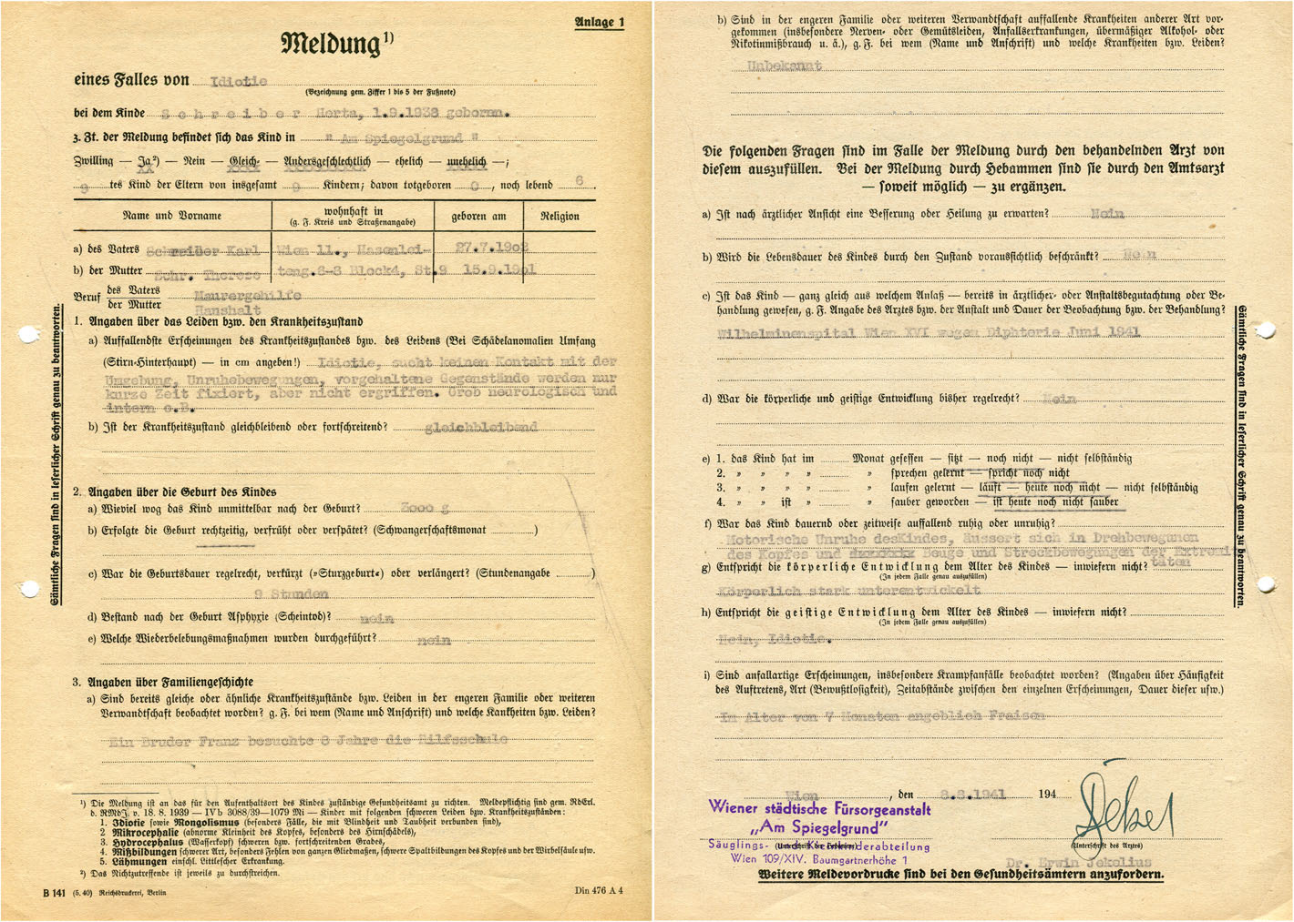
On 8 August 1941, Erwin Jekelius reported Herta to the Reich Committee for the Scientific Registration of Serious Hereditary and Congenital Illnesses, the secret organization responsible for the child “euthanasia” program (WStLA)

Herta was admitted to Spiegelgrund on 1 July 1941. On 8 August, Jekelius reported her to the Reich Committee for the Scientific Registration of Serious Hereditary and Congenital Illnesses, the secret organization behind child “euthanasia.” In the form he sent to Berlin, Jekelius pointed out that Herta had no chance of recovery but that her condition would not curtail her life expectancy—an unacceptable combination in the eyes of the euthanasia “experts” (Fig. 8). On 2 September, a day after her third birthday, Herta died of pneumonia, the most common cause of death at Spiegelgrund, which was routinely induced by the administration of barbiturates over a longer period of time.^96^

**Fig. 9:**
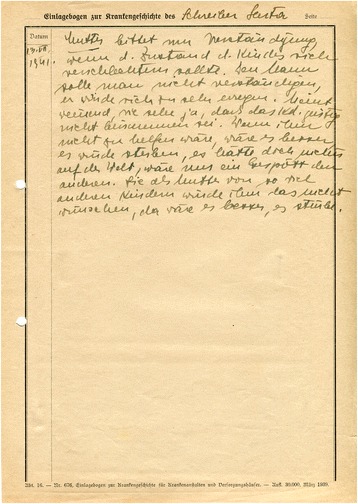
A note in Herta’s Spiegelgrund file suggests her mother was aware that her daughter would be killed at the clinic (WStLA)

A note in Herta’s Spiegelgrund file indicates that her mother not only knew what fate awaited her child at the facility but that she accepted or even expected it:

Mother asks to be notified if the condition of the child should get worse. The husband should not be informed, he would be too upset. She says in tears that she can see for herself that the child is mentally not well. If she cannot be helped, it would be better if she died. She would not have anything in this world, she would only be ridiculed by others. As the mother of so many other children she would not want that for her, so it would be better if she died.^97^ (Fig. 9)

In the context of Nazi-ruled Vienna, it seems that Herta’s mother, with a husband at war and six children to take care of—one of them with a severe mental disability—had reached a point where the possibility of having that responsibility taken off her shoulders would seem like a relief, even if it meant knowingly surrendering her daughter to death. In a society permeated by contempt for “unworthy life,” the social stigma of mental disability must have been acute—and the fear of ridicule is indeed the main argument in the quoted document. From Herta’s religious denomination *gottgläubig* (deistic), it can be inferred that the family had left the Catholic Church under the influence of the Nazis’ opposition to organized religion, a practice that was usually followed only by a radical minority of Nazi sympathizers ([82]: 281–3). To this, we have to add a lack of institutional support since more and more homes for disabled children were dissolved and rededicated as institutions for the “healthy” and “valuable” children.

What took place between Herta’s mother and Asperger before the latter decided to transfer Herta to Spiegelgrund? Did they openly discuss the possibility of “euthanasia”? If so, did she turn to Asperger with her mind already made up, or was it he who offered this as a “solution” to her? Or did he decide independently what he thought best, based on the information she provided? From the available documents, we cannot know with certainty. All we have to go by is Asperger’s short note on Herta in which he calls for her “permanent placement” at Spiegelgrund—whether this was a conscious euphemism for murder or not, it is clear that he did not expect Herta to return.

This case is revealing not least with respect to Asperger’s therapeutic credo. As previously mentioned, he repeatedly called for giving people with mental anomalies the best available care in order to develop their potential as far as possible. However, he never addressed the question of what should happen in cases without hope of improvement. The children Asperger advocated for were those who promised some future benefit to society. We must not confuse them with the group labeled *bildungsunfähig* (uneducable), which was targeted for murder in the child “euthanasia” program. In Herta Schreiber’s case, Asperger did not expect any future improvement, rendering further efforts futile. His diagnosis (albeit with a question mark) was “post-encephalitic status.” In 1944, he published an article on this topic, in which he wrote: “All the work done at our ward is carried forward by a strong pedagogic optimism. But in the case of these post-encephalitic personalities, we too have to say that one in most cases has to largely capitulate” ([83]: 116).^98^ The transfer of Herta Schreiber to the Spiegelgrund facility looks like such a capitulation.

**Fig. 10:**
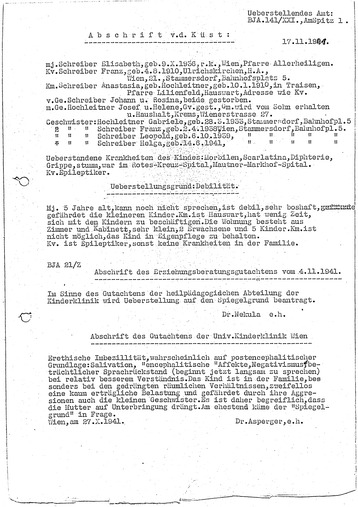
In the case of Elisabeth Schreiber, Asperger also recommended transferal to Spiegelgrund (WStLA, 1.3.2.209.10, Nervenklinik für Kinder, Krankengeschichte Elisabeth Schreiber)

Perhaps it is no coincidence that another girl who was recommended for transferal to Spiegelgrund by Asperger suffered from similar symptoms, also attributed to an earlier infection. According to Asperger’s evaluation, the case of 5-year-old Elisabeth Schreiber (no apparent relation to Herta) bears other similarities as well:

Erethic imbecility, probably on a post-encephalitic basis. Salivation, ‘encephalitic’ affects, negativism, considerable language deficit (is now slowly starting to speak), with relatively better comprehension. In the family, the child is without a doubt a hardly bearable burden, especially under their crowded living conditions, and due to her aggressions she endangers the small siblings. Therefore it is understandable that the mother pushes for institutionalization. Spiegelgrund would be the best possibility.^99^ (Fig. 10)

According to Asperger’s notes, it seems that Elisabeth’s mother was also unable or unwilling to take care of her, but there was no explicit reference to the possibility of her death. What can be said with a degree of certainty is that she sought institutional care for her daughter and that Asperger recommended transferal to the killing facility. However, Elisabeth was not immediately transferred to Spiegelgrund, probably because there was no bed available. Instead, she was sent to another institution for children with mental defects, where she stayed for a few months. In March 1942, she was transferred to Spiegelgrund. One of the nurses wrote that she was friendly and affectionate but that she spoke only one word: “Mama.” She died of pneumonia—like Herta and so many other children at Spiegelgrund—on 30 September 1942, shortly before her sixth birthday.^100^

Was Asperger aware that Elisabeth would have almost no chance of survival at Spiegelgrund, that he was sending Herta to her death? Is it possible that he meant “permanent placement” just in its literal meaning, or do we have to consider it a euphemism for murder (comparable to “special treatment,” “final solution,” or less obliquely, “euthanasia”)? Significantly, the extermination of the mentally ill was never explicitly referred to in written documents, at least not outside the smallest circles of the initiated. For example, Hitler’s 1939 “authorization” providing cover for the extermination of 70,000 people in gas chambers only mentioned the intention to “accord a merciful death” in individual, carefully selected cases ([84]: 114). In documents not protected as state secrets, it would have been a grave breach to even mention the possibility of killing patients. Asperger’s expression, used in reference to a secret killing facility, could hardly be understood as anything other than a recommendation for “euthanasia”—provided that he knew what was going on there.

While the “euthanasia” killings at Spiegelgrund (as elsewhere) were officially a secret, and parents were routinely deceived about the true nature of the institution and the fate awaiting their children, rumors nevertheless abounded, and Asperger was in an exceptional position to know the truth. After his arrest in 1945, Ernst Illing (1904–1946), Jekelius’ successor as Spiegelgrund’s director, gave the following statement:

I point out that my clinic [Spiegelgrund] was always overcrowded, since other clinics […], including the University Pediatric Clinic, transferred—or wanted to transfer—such hopeless cases, evidently because they believed that in my clinic euthanasia was possible on account of the mentioned circular, while they were not allowed to practice euthanasia. I am absolutely convinced that the directors of the mentioned institutions were aware of euthanasia and the mentioned circulars.^101^

Illing had every reason to diminish his own responsibility, but there is further evidence for the close ties between Spiegelgrund and the university clinic. As mentioned before, Spiegelgrund’s founding director Jekelius had trained under Hamburger and Asperger; Jekelius and Asperger were colleagues at the Vienna Public Health Office, and all three men played a leading role in the establishment of the Viennese Association for Heilpädagogik in 1941, part of a broader attempt to strengthen curative pedagogy’s profile in Nazi Germany as a medical discipline in accordance with race hygiene ([4]: 172–3). In line with Illing’s testimony, children were routinely sent from the pediatric clinic to Spiegelgrund ([13]: 203). A number of them were subjected to tuberculosis vaccine experiments by Asperger’s colleague Elmar Türk. After the experiments, the children were sent to Spiegelgrund, where they were murdered so that the vaccine results could be compared with the pathological findings. Staff at the pediatric clinic were not only aware of what happened at Spiegelgrund but exploited the research opportunities created by the murders [85].

Furthermore, knowledge of the mass murders euphemized as “euthanasia” was not limited to insiders; it was in fact widespread among the Viennese population. During the so-called “T4” killing campaign, patients’ relatives staged public protests in front of the Steinhof psychiatric hospital in Vienna. They could not prevent approximately 3200 Steinhof patients from being transported to the gas chamber at Hartheim, but they took a courageous stance against the regime’s campaign of murder.^102^ Rumors were so widespread that the Viennese edition of the *Völkischer Beobachter*—the Nazi Party’s daily newspaper—was compelled to deny the killings. The article mentioned lethal injections and even gas chambers, which shows how specific the public’s knowledge was ([86]: 7). Anny Wödl, a Viennese nurse, had no doubt that the transfer of her son Alfred to Spiegelgrund, enforced in 1940 despite her resolute resistance, would mean his death ([87]: 298). Even abroad, the killings at Spiegelgrund became known. In the fall of 1941, the Royal Air Force dropped leaflets mentioning both the Steinhof hospital and Jekelius’ name in connection with the systematic murder of patients.^103^

In light of this evidence, it seems extremely implausible that Asperger—a longtime colleague of Erwin Jekelius and a well-connected player in his professional field—was unaware of the activities at Spiegelgrund. When he reflected on the Nazi period in 1974, Asperger did not mention the child “euthanasia” program directly but claimed that he had from the outset refused to accept the Nazis’ concept of “unworthy life” or to participate in race hygiene measures, implicitly acknowledging that he was aware of its ramifications [3].

In the cases of Herta and Elisabeth, were there alternatives to sending them to Spiegelgrund? Could he have saved their lives? Under the circumstances, and given the parents’ lack of support, ensuring the long-term survival of the two children would certainly have been difficult. Facilities for children with severe disabilities continued to exist (both public and religious), but they were under pressure to hand over those among their patients deemed “unworthy” of support. Nevertheless, Asperger was under no obligation to send children directly to the killing facility, even if they suffered from severe disabilities. He could, without any risk to himself, have transferred them elsewhere, and in a number of other cases, he did just that.

Among the children who died at Spiegelgrund, there were at least four others apart from Herta and Elisabeth who had previously been examined by Asperger, two of them while the Spiegelgrund “euthanasia” facility was already in operation. Their conditions were so severe that the “euthanasia” apparatus ultimately caught up with them, although Asperger initially had them transferred to other institutions.^104^ Why did he send Herta and Elisabeth to Spiegelgrund, but not Richard and Ulrike? While Asperger’s diagnostic report on Richard (who was diagnosed at Spiegelgrund with “mongolism”) is not included in the surviving records, Ulrike’s file contains a report in which Asperger described her as “mentally extremely retarded, severely autistic,” and as a “severe burden” at home. Over the course of a year, he had observed a process of “cerebral decay” which led him to recommend a home for mentally disabled children.^105^ There is insufficient evidence to determine with certainty why he decided one way or the other, although in the cases of Herta and (less clearly) Elisabeth, the attitude of the parents may have played a role. The evidence in these two cases suggests that at least under the given circumstances he accepted the killing of disabled children as a last resort. This needs to be kept in mind when assessing Asperger’s role in a wave of transferals to Spiegelgrund which resulted in the deaths of a considerable number of children.

In December 1941, the authorities in Niederdonau (the province surrounding Vienna) noticed that patients in the children’s ward at the Gugging psychiatric hospital were not attending school, despite not having been excused.^106^ An expert committee was consequently convened to evaluate the children with regard to their “educability.” Children evaluated as “educable neither in a special school nor within a psychiatric institution” were to be “delivered to the operation of Dr. Jekelius as soon as possible.”^107^ This formulation implies that the recipients of the document would know who Dr. Jekelius was and that the children deemed “uneducable” by the committee should be killed.

Due to overlapping jurisdictions (the Gugging hospital was on Viennese territory and property of the city, but leased to the Niederdonau administration), the committee consisted of seven members from both provinces. Asperger was asked to join in his role as medical advisor for Vienna’s special school system. He was the only expert on Heilpädagogik on the panel and the only clinician with scientific credentials (the only other physician was the director of the Gugging mental institution, the psychiatrist Josef Schicker, 1879–1949).

After 106 children had been transported in March and May 1941 to the killing center in Hartheim, at the end of that year, 220 patients remained on the ward. In the commission’s report, dated 16 February 1942, all children of school age were classified into various categories, with 35 (9 girls and 26 boys) labeled as “uneducable” and “unemployable,” the keywords for “euthanasia.” The report does not include their names, rendering it impossible to establish with certainty what happened to them individually.^108^ However, there is evidence for a number of later transferals from Gugging to Spiegelgrund with fatal outcomes.^109^

On 20 May 1942, 3 months after the commission convened in Gugging, nine boys were transferred to Spiegelgrund. All of them were dead within a few months. By the end of that year, another 20 children (9 girls and 11 boys) followed, only to meet the same fate. During 1943, 12 children (8 boys and 4 girls) were taken to Spiegelgrund, none of whom survived.^110^ The death rate of 100% indicates that these children were sent to Spiegelgrund to die. The time-lapse between the commission’s visit and some of the transferals is most likely due to the fact that Spiegelgrund was routinely running over capacity; it is also possible that in some cases, further observation was considered necessary.

The commission relied on suggestions prepared by Schicker but examined the children individually and took a decision in each case. Among a group of 50 children whom the director deemed unfit for school and wanted to keep in Gugging, the committee found 18 who in their opinion warranted further pedagogical efforts. However, regarding the 35 children placed in the lowest category by Schicker, the commission confirmed his verdict in every case: “The school-age children who are uneducable, incapable of any development or occupation [*nicht bildungs- und entwicklungs- bzw. beschäftigungsfähig*] were examined and it was determined that in none of these cases noteworthy education results could be expected.”^111^ By changing the diagnosis for 18 children of the first group, the commission improved their chances of being sent to a special school rather than remaining at the mental hospital, which means that they faced a lesser risk of being selected for killing. Even so, 20 children of the original 50 ended up as “euthanasia” victims at Spiegelgrund, in addition to the 35 children whose classification as hopeless cases slated for “euthanasia” had been confirmed by the panel. In all, 59 of 158 children evaluated by the commission died at Spiegelgrund before the end of the war, a death rate of 37.3%.^112^

Was the commission in a position to save at least some of the children had they wanted to? Due to the limited sources available, this question cannot be answered conclusively. What can be shown, however, is that at least in some cases their families wanted to take them into their care but were not permitted to do so by the authorities. Engelbert Deimbacher was a patient at the children’s ward when the commission visited. He was deaf-mute from his birth in 1929. His case file mentions hydrocephaly and severe mental disability. Although he could not attend school, there was hope he might be able to improve his physical abilities to perform simple tasks. He was described as lively and sociable. Engelbert’s file contains three letters from his father asking him to be released into his care, the last of which was received on 15 February 1942, 3 days before the commission’s visit. The requests were denied on all three occasions, the last under the pretext that further examinations were necessary. On 20 May 1942, Engelbert was transferred to Spiegelgrund, where he died on 8 November.^113^ In the case of Georgine Schwab (born 1934), her grandmother repeatedly pleaded for her release, again to no avail.^114^ The files contain numerous similar examples, proving that these children were neither unwanted nor unloved.^115^

In this case, it seems that Asperger was a well-functioning cog in a deadly machine. Even if the ultimate responsibility for the deaths of these children fell on Schicker, Gugging’s director, who signed off on the transferals, and on the Spiegelgrund staff, the episode shows that the authorities trusted Asperger to lend his expertise to the selection of children for elimination.

### Asperger’s diagnoses compared to those at Spiegelgrund

In his publications, Asperger projected an image of himself as benevolent, optimistic, and affectionate towards the children in his care—a characterization echoed in the biographical literature. While there is little doubt that he was passionate about his work and genuinely cared about many of his patients, in the context of this paper, we must ask whether this positive attitude extended to those children who did not offer hopes of future development or who defied attempts to educate or discipline them. Based on the narrative promoted by Asperger himself and others who took his cue, one would expect to find considerable differences between his reports on troubled children and those written by colleagues committed to the idea of “unworthy” lives and their exclusion from the body politic.

The records of 46 children who were examined both by Asperger at his Heilpädagogik Ward and at Spiegelgrund allow this to be put to the test; of these 46 children, 6 died at the “euthanasia” facility; their cases, including those of Herta and Elisabeth Schreiber, are discussed above. The following analysis focuses on the remaining 40 children (12 girls, 28 boys), who survived Spiegelgrund and were later transferred to other institutions or discharged.^116^ In ten of these cases, Asperger explicitly called for transferal to Spiegelgrund, and in four, he recommended an “institution under curative pedagogic leadership,” which also points to the Spiegelgrund.^117^ Although other instances—especially the Youth Welfare Administration—were also involved in determining what would happen to the children, Asperger was the leading expert in the field, and his diagnostic reports and recommendations were often decisive.

Unlike with Herta and Elisabeth Schreiber, in the 14 cases in question, there is no indication that Asperger expected the children he recommended for transferal to Spiegelgrund (explicitly or by suggestion) to be killed there. Although the Spiegelgrund facility was established to implement the child “euthanasia” program, it also carried out long-term observation of children with developmental or other problems, housed infants with less severe disabilities, and also served as a disciplinary facility for the youth welfare system [81]. The conditions of these 14 children appear not to have been so severe as to make them targets for extermination, although sending them to Spiegelgrund nevertheless put them at considerable risk. According to the survivors’ testimony, children were routinely subjected to violence, including medicalized forms of torture, and the older ones lived in fear of being killed.^118^

The sample of 40 Spiegelgrund survivors previously examined by Asperger includes 30 cases with sufficient documentation to allow a comparison between Asperger’s evaluations and those of his colleagues who were directly involved in the murder of disabled children (the cases excluded from the direct comparison due to insufficient documentation include Friedrich K., who fit the profile of “autistic psychopathy”^119^). Is there any evidence in these files that Asperger attempted to draw a positive picture of the children in order to minimize the risk they faced from the Nazis’ race hygiene policies? To be sure, the direct comparison raises certain problems: The assessments varied in length and depth, they did not adhere to common diagnostic standards, and sometimes considerable time passed between them so that the children’s conditions could have evolved in the meantime, for better or for worse. Despite these limitations, the files represent a unique opportunity to assess Asperger’s work as a diagnostician within the institutional and methodological context of his time and place. Spiegelgrund, established not only for child “euthanasia” but also for dealing with “difficult” or “asocial” children, epitomizes the implementation of race hygiene in pediatrics, youth psychiatry, and youth welfare. The senior staff at Spiegelgrund (who were the authors or signatories of the medical reports analyzed here) were committed Nazis and race hygienists. Against this background, any systematic bias Asperger might have had in favor of his patients would have to be visible in this sample. And yet, out of these 30 cases, there are only 2 in which Asperger appears to judge the children less harshly than his peers at Spiegelgrund. In 16 or just over half of the cases, Asperger and the diagnosticians at Spiegelgrund came to comparable conclusions. In the remaining 12, Asperger took a more negative and in some instances an outright disparaging view of his patients.

Gerald St. is the second boy in the sample who besides the aforementioned Friedrich K. was described, among other labels, as “autistic.” Asperger saw him in July 1941, when he was 28 months old. He diagnosed him with “intellectual retardation” and a “disturbed personality,” and more specifically with a “restriction of personal contact, abrupt impulses, increased and inadequate affects, and stereotypical movements.” In the context of a “normal children’s community,” he considered the boy an “unbearable burden” and therefore recommended either private care or transferal to Spiegelgrund.^120^ Gerald was admitted to Spiegelgrund 8 months later, via two other institutions. The first psychological assessment at Spiegelgrund came to similar conclusions: “intellectually retarded, especially with regard to language,” “impulsiveness,” and “tendency to tantrums.” “It is very difficult to establish contact, the child just talks in a spontaneous and autistic manner.” The overall diagnosis was “neuropathy.”^121^ A year later, Heinrich Gross (1915–2005), one of the most notorious Austrian “euthanasia” perpetrators, came to a much more optimistic result and recommended Gerald’s release into the care of his grandparents as Gerald, although still behind in his overall development, had caught up regarding his mental abilities. Gross now described the boy as emotionally responsive, cheerful, and excitable.^122^ This is an example of Asperger’s reputed “pedagogical optimism” ringing hollow in the face of what he actually wrote in his patients’ files.

Gerald was initially described in similar terms by Asperger and at Spiegelgrund. Leo A., by contrast, is a typical example of the 12 out of 30 cases in which Asperger appears harsher than his peers. Born in April 1936 to a single mother, Leo was placed in foster care immediately after his birth. At age four, Leo was an intelligent but difficult child. He suffered from fits of rage and was accused of cruelty towards animals. In November 1940, he was sent to Asperger’s ward for observation and diagnosis. In his assessment, Asperger qualified Leo as a “very difficult, psychopathic boy of a kind which is not frequent among small children.” Although he was “in some respects intellectually ahead of his age,” Asperger pointed out the boy’s “heightened impulsiveness” and his “acts of malice carried out with great skill.” Asperger’s recommendation contains an expression he often used to characterize his ideal style of education: What the boy needed was the “very sovereign guidance” (sehr überlegene Führung) that only an institution following the principles of Heilpädagogik (such as Spiegelgrund) could provide.^123^ Leo was sent to Spiegelgrund 4 months later, after a stay with his aunt. After 4 months of observation, Erwin Jekelius and Heinrich Gross signed their own assessments: Leo was “very well developed in every respect and very intelligent.” He was found to be solitary and withdrawn in the company of other children and easily irritated, but he caused no difficulties. While not very helpful towards other children, no signs of a lack of empathy (*Gemütsarmut*) had been observed. Jekelius’ and Gross’ recommendation was to return the boy to his father since they thought that the difficulties leading to his hospitalization had been caused by his environment in foster care. Asperger’s diagnosis of “psychopathy”—with its implication of a constitutional, potentially lifelong condition—had no merit in the eyes of his former collaborator Jekelius.^124^

In this as in other cases, Asperger’s belief in the etiological preponderance of innate constitutional factors (or, alternatively, organic brain damage) led him to negative verdicts on his patients, which could easily turn into self-fulfilling prophecies.

Asperger’s report on another 4-year-old, Karl E. (like Leo a foster child), is similarly harsh and devoid of any discernible positive bias when measured against the diagnoses produced at Spiegelgrund. Asperger characterized him as “a psychopathic infant who causes considerable pedagogic difficulties: marked irritability […], a tendency towards negativistic reactions and acts of malice, demanding character.” He recommended transferal to a closed institution as the only viable possibility for the boy, conceding that in this case, the boy had potential thanks to his intelligence.^125^ After several months of observation at Spiegelgrund, Jekelius concluded that “contrary to the assessment at the pediatric clinic, the diagnosis of psychopathy could not be confirmed.” The boy’s behavior was not outside the normal range: He was “very intelligent” and “solved with ease” the questions and puzzles put to him by the psychologist.^126^

The case of 16-year-old Johann K. illustrates Asperger’s tendency to downplay the importance of the children’s circumstances (including instances of mistreatment and abuse) and to explain difficulties they may have experienced (or caused to caregivers) with alleged constitutional deficiencies. Asperger called Johann a “semi-imbecile,” although he conceded that his achievements at school were not that bad considering he had missed years of school because of bone tuberculosis. Asperger saw the main problem in the boy’s “severe irritability and lack of inhibition in every respect (severe aggressions, sexual over-excitability, prodigality, laziness).” Provided that he was placed under “very sovereign, inexorable guidance,” Asperger thought it possible that Johann could be used for unskilled labor. Left with his parents or grandparents, Asperger considered the boy a “danger to his environment” who would without a doubt end up “in total neglect.” He recommended removing the boy from his family and referring him to a closed institution.^127^ For unknown reasons, Johann was not sent to the institution recommended by Asperger, but to Spiegelgrund. Ernst Illing, Jekelius’ successor, concurred with Asperger that the boy’s intellectual development was lacking. By contrast to Asperger, Illing pointed to an alleged “hereditary burden” based on his mother’s moral conduct and raised the possibility of sterilization. And yet, Illing’s assessment of Johann’s character remained more optimistic than Asperger’s: In his view, the main problem had been a lack of “pedagogic encouragement”; despite his difficult childhood, the boy suffered from “no major abnormalities,” apart from a lack of initiative that Illing attributed to his lengthy hospital stays. He also saw no need for institutional care, recommending instead placement with a foster family in one of the “rural suburbs of Vienna.”^128^

Another example of Asperger’s tendency to downplay the consequences of neglect or abuse are his comments on two sisters of 7 and 5 years, whom he saw in February 1941 because their mother had difficulties with them. He wrote that Charlotte (the younger one) was “more severely degenerative than her sister,” “intellectually clearly retarded,” and “always ready for serious mischief,” The mother, whom he characterized as “not very intelligent and mentally slightly strange,” was in his view not able to cope with the two girls, requiring their immediate placement in a closed institution.^129^ Illing’s conclusion on Charlotte, by contrast, stressed that she had spent the first years of her life in institutions and in foster families and that her mother had severely neglected her when she took custody. Where Asperger had seen signs of “degeneration,” Illing squarely attributed Charlotte’s difficulties (and her slight “mental retardation”) to the neglect she had experienced, although he also pointed to alleged hereditary deficiencies in her family.^130^

As mentioned, of these 30 cases, there are only 2 in which Asperger appears to have taken a more positive position than his peers at Spiegelgrund: In November 1938, he saw 6-year-old Johann T., whom he described as “an erethic, feeble-minded boy who recognizes no danger and who, unless constantly supervised, due to his restless drivenness endangers himself and his surroundings.” Asperger recommended institutionalization at the Biedermannsdorf reformatory near Vienna (Spiegelgrund had not been established yet).^131^ At Biedermannsdorf, as in similar institutions, children were routinely subjected to emotional, physical, and sexual violence from their peers and from staff [88]. It is hardly surprising then that Johann did not make much progress over the next years. In May 1941, Jekelius diagnosed the boy as “uneducable” and an “imbecile” and demanded his transferal to Spiegelgrund. Despite the dangerous diagnosis, Johann survived the “euthanasia” facility, though his later fate remains unknown.^132^ Asperger’s diagnosis in this case appears more lenient and optimistic, but it is possible that Johann’s state deteriorated during the 30 months between the two diagnoses, especially in light of the adverse conditions at Biedermannsdorf.

The second case is similarly inconclusive. In October 1940, Asperger saw the 16-year-old Hildegard P. because her promiscuous lifestyle had aroused the authority’s suspicions. Despite describing her in unflattering terms (“not many inhibitions in sexual regards,”) he recommended releasing Hildegard into the care of her mother but placing her under close surveillance by the National Socialist Welfare Organization (NSV).^133^ Seven weeks later, Jekelius decided to institutionalize Hildegard on the grounds of her “sexual depravation.” Although there are many examples in which Asperger had no qualms committing girls to closed institutions on similar grounds, in this case, he showed more leniency. For Hildegard, it meant the difference between freedom and confinement to a reformatory.^134^

The cases analyzed here demonstrate that Asperger did not refrain from diagnoses such as “feeble-mindedness,” which could entail serious dangers in the context of a youth welfare system dominated by an eliminatory ideology towards the weakest members of society. In one regard, however, Asperger did show a certain restraint. As outlined in the previous section, while the Spiegelgrund staff routinely included information on the patients’ and their families’ “hereditary qualities” and sometimes even raised the possibility of (forcible) sterilization, Asperger in most cases avoided such references.

Apart from this qualification, the sample yields no evidence that Asperger proved more benevolent towards his patients than his peers at Spiegelgrund when labeling children with diagnoses that could have an enormous impact on their future—quite the opposite. Like many of his colleagues, Asperger had a marked tendency to separate children from their families—which he often considered dysfunctional—and to commit them to closed institutions. Of course, many children were exposed to violence or neglect at home, and institutional education in principle could have been a means of protecting them. All too often, however, it seems that Asperger preferred the pedagogical environment of a hierarchical institution over the home provided by parents he considered neurotic, incapable, or merely too “weak” in dealing with their child. In practice, though perhaps despite his best intentions, this meant that he regularly sent minors to institutions ripe with abuse and violence [89].

In 1941, Asperger sent a 15-year-old boy to a “labor education camp for work-shy youth” in Bavaria because he hoped that strict discipline and forced labor would help alleviate his severe hypochondriac symptoms.^135^ Although this case is in some respects unusual, it illustrates how authoritarian Asperger’s approach could be. The case records kept by his clinic are full of examples revealing how he considered strict discipline and “sovereign guidance” (*überlegene Führung*, a signature phrase in his written reports) the answer to many of his patients’ (and their caregivers’) troubles.^136^

### Asperger in the post-war years

This is not the place to give a full account of Asperger’s post-war career, which spanned more than three decades, therefore I will limit myself to a few points that are relevant in the context of this paper. Little is known about Asperger’s life during the final 2 years of the war, which he spent in the Wehrmacht. After 9 months of training and service in Vienna and Brünn/Brno, he was sent to Croatia in December 1943 with the 392nd Infantry Division, deployed for “protection” of the occupied territories in Yugoslavia and the fight against “partisans.”^137^ The German forces’ tactics against irregular troops in Yugoslavia included mass killings of civilians as hostages or in reprisals, resulting in tens of thousands of deaths ([90]: 161). Asperger briefly mentioned his war experiences in his 1974 interview:

[…] I was in the war, I was deployed in Croatia in the anti-partisan war… I would not like to miss any of these experiences. It is good that a man knows how he behaves in mortal danger, with the bullets whistling. It is also a proving ground. And a ground where one has to care for others. It is also a great gift from destiny that I never had to gun anybody down [3].^138^

After the defeat of Nazi Germany, Asperger returned to the Vienna University Pediatric Clinic. The Heilpädagogik ward had sustained severe damage from a bomb attack which also killed Viktorine Zak, Asperger’s closest assistant [3]. On 1 September 1945, Asperger applied for the confirmation of the Habilitation he had obtained in 1943—all such degrees awarded during the Nazi period were made void upon liberation, pending an inquiry into the candidate’s political background. As mentioned, in 1938, Asperger had joined the National Socialist Welfare Organization (NSV) and the German Labor Front (DAF) and had applied for membership in the National Socialist German Physicians’ League (NSDÄB).^139^ In contrast to party formations such as the SS or Hitler Youth, these were considered “affiliated organizations” of the Nazi Party, and not part of the NSDAP itself. This distinction allowed Asperger to emerge with a clean slate under the Austrian implementation of denazification since he had never joined the NSDAP. He avoided the career interruptions that many of his colleagues faced and retained his position as the head of the Heilpädagogik ward.^140^ Additionally, from July 1946 to May 1949, he served as provisional director of the pediatric clinic. In 1957, he moved to Innsbruck, where he headed the local university pediatric clinic until 1962 when he was formally appointed as Chair of the Vienna Pediatric Clinic, the most prestigious position in Austrian pediatrics.^141^

With respect to Austria’s Nazi past, judging from his writings, Asperger formed part of the wall of silence established during the first years after the war. He made a rare reference to the Nazi period in his 1977 retirement speech from the Vienna clinic, vaguely referring to the Germans’ “arrogance, hubris, [and] cruel iniquities” which had “inexorably led to war” and to “terrible suffering.” As in his 1974 interview [3], he painted the war in terms of his personal experiences as an existential learning opportunity ([4]: 196, [91]: 217). According to some, Asperger in 1938 risked his life to speak out against the threat that race hygiene ideology posed to the children in his care. In 1977, while explicitly addressing the war in a speech summarizing his intellectual legacy, he did not care to mention National Socialism, its millions of victims, or even the hundreds of children, some of them his patients, who had been killed practically under his eyes.

Although later in his career he represented pediatrics as a whole, Heilpädagogik remained his central concern. At least in Austria, he dominated the field for decades, curtailed only by competition from the emerging discipline of youth psychiatry.^142^ Judging from his writings after 1945, the central tenets of his thinking and his pedagogical approach remained relatively unchanged. On a conceptual level, he saw his main opponents in the representatives of psychoanalysis and related theories focusing on dynamic psychological processes and childhood experiences ([76]: 2–3, 272, and numerous other passages). In principle, he also distanced himself from the genetic determinism typical of Nazi race hygiene, at least to the extent necessary to claim a space for his own discipline and its therapeutic options ([76]: 55). Yet despite his often-stressed “pedagogic optimism,” he believed that his patients were a “selection of children with endogenous constitutional damages” ([76]: 79). It is hardly surprising, then, that he would refer to his work as a heroic and often hopeless fight against the terrible odds of constitutional deficiencies of all kinds ([76]: 272–5). A typical example of his approach is a 1952 paper on the “Psychopathology of Young Criminals,” in which he named three groups of children with constitutional or organic defects as particularly prone to committing crimes: the so-called “unstable” (or “disorganized”) type, those with encephalitis-induced brain damage, and the “autistic, with disturbed instincts, especially those with normal or above-average intelligence” ([92]: 31).

Despite his emphasis on heredity and constitution, he mostly avoided explicit references to eugenics, which due to its association with Nazi crimes had become discredited in mainstream scientific discourse, at least in Austria and Germany. In one passage of his textbook, he criticized the term “unworthy of living” and stressed the need to dedicate the best schools and the best teachers to the education of the mentally disabled ([76]: 93). However, in a book with very few references overall, he also quoted Otmar von Verschuer (1896–1969), one of the leading race hygienists in Nazi Germany with ties to Josef Mengele [93, 94], and Johannes Lange (1891–1938), a contributor to the Nazis’ “Bible” of race hygiene [95]), using their twin research to bolster his views on the importance of heredity ([76]: 53–4, 140, 144, 207, 274). In his handbook on Heilpädagogik, Asperger also included the following passage on the eugenic dangers of “feeble-mindedness”:

Multiple studies, above all in Germany, have shown that these families procreate in numbers clearly above the average, especially in the cities. [They] live without inhibitions, and rely without scruples on public welfare to raise or help raise their children. It is clear that this fact presents a very serious eugenic problem, a solution to which is far off—all the more, since the eugenic policies of the recent past have turned out to be unacceptable from a human standpoint ([76]:88).^143^

While eugenics appeared only of peripheral concern to Asperger, the idea of an inherited “general inferiority of the nervous system” as a common etiological basis for most childhood disorders was of central importance to him ([76]: III, 1–3, 53–61, 272). In a number of passages, this is linked to the concept of “degenerative stigmas”—small bodily anomalies, which were supposed to indicate the “degenerative constitution” of some of his patients ([76]: 84, 85, with a reference to Lombroso, 86–7, 125, 142, 194).

One troubling consequence arising from this approach is how Asperger regarded the sexual abuse of children. He was convinced that victims of sexual abuse shared a common constitutional disposition and certain character traits such as “shamelessness,” leading them to “attract” such experiences, while children with “natural defensive forces” should be able to “reject” them ([96]: 27).^144^ If a child suffered from trauma as a result of abuse or rape, Asperger again took this as a sign of an inherent constitutional weakness, since a “healthy personality” should be able to “outgrow” even “brutal acts of sexual violation” without suffering any damage in terms of psychological development ([96]: 24, [76]: 58–60, 197, 262–3). In his textbook, the only examples offered on this subject are cases in which the abuse was presented as a fabrication of the child, reinforcing the impression that the victims were always to blame—either because they were phantasizing, if not outright lying, or because they had “provoked” the deeds due to their constitutional predisposition ([76]: 233, 250–6).^145^

The case of 15-year-old Edith H. illustrates the continuity of Asperger’s thinking on sexual abuse from the Nazi to the post-war period. Edith was admitted to the Heilpädagogik ward in April 1941 because she had been sexually abused by a 40-year-old man. In his report, Asperger called her “under-developed with regard to intellect and character.” He deplored that she lacked “moral sense” and did not show any remorse about what had happened. He recommended placing her in permanent welfare care (*Fürsorgeerziehung*), not just because of her “severe sexual depravation” but also because of the moral danger she allegedly posed to her environment. A few months later, in accordance with Asperger’s recommendation, the court ordered her forced admittance to Spiegelgrund. During her stay, according to the physician Helene Jokl and Spiegelgrund’s director Erwin Jekelius, she was friendly, helpful, and comradely, but also lazy and susceptible to both positive and negative influences. In contrast to Asperger, they considered her intelligence average, but echoed his opinion on Edith’s “sexual depravity.” They recommended sending her to Theresienfeld, a reformatory for girls.^146^

In a similar vein, Asperger rejected the possibility that constitutionally healthy children could suffer from war-related trauma. Any observable symptoms were again either due to some inborn constitutional defect or arose from the desire to gain material advantages, such as pensions ([76]: 141, 194).^147^ The case of Max G. is an example of the impact this narrow focus on a child’s alleged “constitution” could have on their lives. In 1938, when Max was 6 years old, his family was torn apart by the Nazis’ anti-Jewish policies. His Jewish father was forced into a divorce and spent 5 years in a concentration camp. With his mother, Max then moved to Znojmo, a town annexed from Czechoslovakia after the 1938 Munich Agreement, from where he was expelled along with the German-speaking population in 1945. At 14 years old, he was living in war-torn Vienna with his father. In August 1946, Asperger wrote an expert opinion for the Juvenile Criminal Court on Max, who was accused of a series of thefts. Not a single word in his assessment referred to the fate of the boy’s father or to the fact that as a “half-Jew” he had himself been under threat of persecution for half his life. While other documents in the file stressed that the boy had finished school with good grades despite his difficult situation, Asperger described him as “intellectually clearly deficient.” Based on the boy’s apparent “overfamiliarity” and “unreliability,” he diagnosed him as an “epileptoid psychopath,” a condition he described as the opposite of “autistic psychopathy” with respect to social behavior. In November 1946, after Max was fired from an apprenticeship that was seen as his last chance to prove his worth, based on Asperger’s diagnosis and recommendation, the boy was sent to the Eggenburg reformatory.^148^

As in other countries, the Austrian public has over the last years been confronted with a wave of revelations on the violence, abuse, and neglect pervading institutions set up to protect children from precisely such conditions [74, 97–103]. The same is true for children with disabilities, who were often kept in asylum-type institutions where they were denied rehabilitation or therapy and exposed to severe hospitalism [104, 105].^149^ In this context, a critical assessment of Asperger’s brand of Heilpädagogik with its “pronounced dominance of restrictive pedagogical concepts” ([74]: 611) is overdue. Specifically, what needs to be investigated is how the ideas he promoted of “hereditary constitutions” as the root of most mental troubles, his bias against victims of sexual and other abuse, his unwavering belief in the benefits of closed educational institutions, and his emphasis on the authority of the “genius educator”—the ideal of a towering father-figure that he had created for himself—impacted the lives of thousands of children who were often stigmatized with the label of “constitutional defectiveness” on scientifically dubious grounds and institutionalized.

## Conclusions

The aim of this paper is to provide a factual basis for the debate on Hans Asperger’s career in Vienna during the Nazi period. The main conclusion is that the narrative of Asperger as a principled opponent of National Socialism and a courageous defender of his patients against Nazi “euthanasia” and other race hygiene measures needs to be revised in light of the examined evidence. What emerges is a much more problematic role played by this pioneer of autism research and the namesake of Asperger’s syndrome. Kondziella, in his 2009 paper on neurological eponyms with roots in the Nazi period, ascribed an “ambivalent role” to Asperger, classifying him as neither a “perpetrator” nor a “protestor” ([11]: 59). This broad categorization^150^ must be re-evaluated now that we have a basis for a much more detailed and evidence-based assessment of Asperger’s problematic role during this dark time.

Asperger’s choices after Austria’s Anschluss to Nazi Germany are best understood against the backdrop of his political socialization during his early years in the Bund Neuland, an organization combining both Catholic and Pan-German *völkisch* ideology. In the years before March 1938, the Bund became a Trojan Horse for illegal Nazi activists. While there is no evidence that Asperger actively supported Nazism before 1938, there was a common ideological ground, as he himself acknowledged after the war. The formative years he spent in an organization that often acted as a bridge between Catholic and Nazi circles help explain how Asperger could launch his career at the Vienna Pediatric University Clinic in 1931, at a time when its newly appointed director Franz Hamburger, a staunch Nazi, began pushing out the clinic’s Jewish assistants and reorienting the institution according to his worldview.

After the Anschluss, like many Austrians who had not actively participated in the Nazi movement during the time it was banned (1933–1938), Asperger tried to acquire political credentials by joining a number of organizations affiliated with the Nazi Party. Unlike his colleagues at the pediatric clinic, however, he did not join the NSDAP or one of its paramilitary formations (such as the SA or SS). This decision did not hurt his career; he could afford to avoid the ideological commitment of party membership thanks to the protection provided by his mentor Hamburger, one of the Nazi figureheads in the Vienna Medical Faculty.

During the following years, repeated assessments of Asperger’s political reliability show that the Nazi authorities saw him in an increasingly positive light, including as someone willing to go along with their ideas of race hygiene. As late as 1943/1944, when seeking approval of his postdoctoral thesis (the text on “autistic psychopaths” that later made him famous), he received the Nazi hierarchy’s consent. Overall, during the years of the Nazi regime, Asperger managed to extend his professional activities beyond his university position, mostly within the Vienna municipal administration and the juvenile court system*.* The exclusion of Jewish doctors, psychologists, and pedagogues from their professions opened new opportunities for those in the field who were not affected by anti-Jewish legislation or political persecution. Apart from some initial reservations due to his Catholic orientation, there is no evidence that the Nazi authorities considered Asperger an opponent of their race hygiene agenda (or to their policies more generally) or that he ever faced reprisals such as the alleged attempts by the Gestapo to arrest him. A plausible origin of this account is the fact that a long-time associate of Asperger’s, Josef Feldner, saved a Jewish boy by hiding him in his home. The way Asperger referred to this episode long after the war suggests that Feldner’s heroic act and the risk of discovery by the authorities made Asperger fear for himself, which would explain his volunteering for military service.

Asperger’s own attitude towards Jews appears ambiguous. As a member of Neuland, he at least tacitly accepted the organization’s anti-Semitic tendencies expressed both in religious and racist-*völkisch* terms. Case records of his Jewish patients show that Asperger had an acute sense of their religious and “racial” otherness and that anti-Semitic stereotypes sometimes found their way into his diagnostic reports. After the Nazi takeover in Austria, the way he pathologized some children’s mental troubles, rather than acknowledging the reality of the persecution they faced, suggests a certain indifference towards their fate under the regime’s anti-Jewish policies. At the same time, his relationships with Jewish colleagues indicate that he separated the anti-Semitic prejudices pervading the social and political spheres in which he was moving from his personal relations—not an uncommon phenomenon in the history of anti-Semitism.

After March 1938, in line with his acquisition of political credentials by joining organizations affiliated with the Nazi Party, he used lectures and publications to signal his fundamental accordance with the Nazi state’s programs concerning race hygiene and public health. At the same time, he called for the necessary resources to take care of the troubled or “endangered” children in need of Heilpädagogik’s support. Although these statements deviated from the hard core of race hygiene ideology with its inhuman devaluation of the “hereditarily inferior,” there is no indication that they were perceived as critical of Nazi policies, as some authors have claimed. Rather, Asperger’s ideas about Heilpädagogik’s mission within the Nazi state, with his emphasis on turning troubled children into useful members of the German body politic, were shared in many circles at the time. Given that Asperger used the same arguments after the war, there is no indication that the utilitarian logic of social worth he used to advocate for his patients—children considered difficult but who sometimes had normal or even above-average intellectual abilities—was merely a rhetorical strategy. Also, it would be a misunderstanding to assume that the small subset of his patients he diagnosed as “autistic psychopaths” benefited as a group from the fact that he considered some of them of superior intelligence. Just as with other diagnoses, everything depended on where they fell on the spectrum of intellectual and other abilities.

The real litmus test for Heilpädagogik under National Socialism was not how it treated the children with potential—in a time of increasing labor shortage, it was hardly controversial that they should be integrated into the “people’s community” and contribute to the war effort—but those with disabilities so severe that from a utilitarian standpoint all efforts seemed futile. Since long before the Nazi period, Heilpädagogik had excluded children with severe disabilities from its remit, leaving them to psychiatric asylums or similar institutions. Overall, Heilpädagogik claimed to be able to salvage those who could be salvaged and to know where to draw the line.

Despite his advocacy, Asperger left the decisive question unanswered: What should happen with those who could not be helped by pedagogic, therapeutic, or medical means? Regarding these so-called “uneducable” children—who faced the greatest threat from the Nazis’ race hygiene policies—Asperger’s promises to turn his patients into valuable members of the “national community” proved futile.

With regard to such seemingly “hopeless cases” of mental disability, the records of Herta and Elisabeth Schreiber suggest that, at least under the circumstances, Asperger was willing to accept the killing of children as a last resort. In Herta’s case, it seems that the mother consented to Asperger’s decision to refer her directly to Spiegelgrund. According to Elisabeth’s medical file, her mother also pushed for institutionalization, although there is no evidence that she knew what fate awaited her child.

Asperger’s involvement in the selection of victims for the child “euthanasia” program includes an episode when, in 1942, he was part of a commission tasked with the screening of more than 200 residents of a home for children with mental disabilities in Gugging near Vienna. The commission’s mandate was to categorize the children according to their intellectual abilities and prognoses and to define a residual group of “uneducable” children who should be killed at Spiegelgrund. Thirty-five children were placed in this group and later died at the “euthanasia” facility. While Asperger was not directly responsible for their deaths, this episode nevertheless shows to what extent he cooperated with the regime’s murderous policies. His role on the commission was linked to his part-time employment at the City of Vienna’s Public Health Office, an additional professional role he had voluntarily taken on in 1940. Cooperation with the “euthanasia” program was by no means obligatory since the operation was illegal even by the standards of Nazi Germany.

The great majority of Asperger’s patients, however, was not threatened by the child “euthanasia” program—they were not mentally disabled, but simply considered “abnormal” or “difficult” in some way. The diagnostic labels they received at Asperger’s clinic, while not life-threatening, nevertheless carried heavy consequences for them. Asperger’s and his colleagues’ opinions determined to a large extent whether a child would be taken from their family and put into foster care or even sent to a reformatory—institutions that were rife with abuse. A comparison between Asperger’s diagnostic practices and those of his peers at Spiegelgrund (regarding, it should be noted, a group with more severe difficulties than Asperger’s average patients) reveals that Asperger’s reports on these children were in many cases harsher in how he described their intellectual abilities, their character, and their future potential than those written at Spiegelgrund. These documents do not support Asperger’s self-professed “pedagogic optimism” or his alleged benevolence towards his patients—quite the opposite.

On the other hand, he appears to have been less inclined to directly invoke the possibility of hereditary defects, which could have justified interventions such as forced sterilization. The case records from his ward contain very few references to the sterilization program or other race hygiene measures, suggesting that he was reluctant to report his patients to the authorities for these specific purposes. However, the fact that in some cases he did provide information to the authorities tasked with the implementation of race hygiene suggests that he did not fundamentally oppose these policies. This is also in line with his public comments on the sterilization law, in which he argued for its necessity, but called for “responsible implementation.” Overall, the importance of this point should not be overstated because the sterilization law in Austria was implemented much later, and on a smaller scale than in Germany, many doctors and hospitals neglected to report their patients without any consequences, and children were not the main focus of the program. There is no evidence that Asperger deviated from the official position of the Nazi state on sterilization, which in this case had decided—at least in principle, and more so in Austria than in Germany—to institute mechanisms of due process for its implementation.

After the war, on the few occasions when Asperger publicly commented on National Socialism, he vaguely criticized “excesses” or moral failings, but did not address the reality of persecution, violence, and destruction wrought by the Nazi regime, especially against the Jewish population. In this unwillingness to deal with the past, he was typical of large segments of Austrian post-war society. In his professional field of Heilpädagogik, which he came to dominate during the three decades after World War II, this had detrimental consequences, as children from difficult backgrounds continued to be labeled as “constitutionally defective” and to be sent to closed educational institutions where abuse was rampant [97, 98].

An overall appraisal of Asperger’s place in the history of youth psychiatry and Heilpädagogik and as a pioneer of autism research will have to go beyond the focus of this paper, which despite the importance of the Nazi period for understanding Asperger’s life and work cannot replace a long due biography. Regarding Asperger’s contributions to autism research, there is no evidence to consider them tainted by his problematic role during National Socialism.^151^ They are, nevertheless, inseparable from the historical context in which they were first formulated, and which I hope to have shed some new light on. The fate of “Asperger’s syndrome” will probably be determined by considerations other than the problematic historical circumstances of its first description—these should not, in any case, lead to its purge from the medical lexicon. Rather, it should be seen as an opportunity to foster awareness of the concept’s troubled origins.

## Abbreviations

BAB: Federal Archives Berlin
CDSB: Christian-Social Student Union
DAF: German Labor Front
DÖW: Documentation Center of the Austrian Resistance
NÖLA: Lower Austria Provincial Archives
NSDÄB: National Socialist German Physicians’ League
NSDAP: National Socialist German Workers’ Party
NSV: National Socialist People’s Welfare Organization
ÖStA: Austrian State Archive
UAW: Vienna University Archives
WStLA: Vienna City and State Archives

## Footnotes

1 A large part of the work for this article was completed at the author’s previous institution, the Documentation Center of the Austrian Resistance (DÖW), Vienna.

2 The postdoctoral thesis as submitted in 1942 has not been found. However, there is no indication that it differed substantially from the 1944 paper.

3 In 1998, Schopler used Asperger’s ties to the German Youth Movement to suggest a bias in Asperger’s original sample in order to undermine his work on autism ([106]: 387). The significance of the Youth Movement in shaping Asperger’s political and ideological orientation is analyzed in the “Asperger’s political background before 1938” section; there is no verifiable connection to the composition of Asperger’s patient population, however.

4 Uta Frith’s translation deviates from the original in at least two respects: She omitted the “general and somewhat discursive” introduction and chose to bring Asperger’s language “into line with current terminology,” using “autism” and “autistic personality disorder” where Asperger had written “autistic psychopaths” or just “personality disorder” ([7]: 37). For example, in the description of Hellmuth, Asperger’s “die ganze Persönlichkeitsstörung, die hier besteht” in the translation becomes “his autism” ([7]: 67; [2]: 111). Frith also changed the title of the paper, replacing “autistic psychopaths” with “autistic psychopathy.” These semantic shifts are of little consequence for most readers, but they compromise the reliability of the translation for a historical analysis of the early evolution of the autism concept, for which the influence of Eugen Bleuler's work on Asperger was central.

5 One of the passages quoted by Gröger to support the claim of Asperger’s “critical, differentiated attitude” is ([2]: 128), where Asperger asserts that autism does not follow a simple hereditary pattern. For a critical discussion of this and other arguments quoted in this literature review, see the “The best service to our *Volk*: Asperger and Nazi race hygiene” section, in which I analyze Asperger’s stance on race hygiene.

6 The source cited as evidence, an online publication by Colin Müller, accessed on 4 July 2006, is no longer available. A text by Müller under the same title available at http://autismus-kultur.de/autismus/geschichte/hans-asperger.html seems to have been changed, as it contains no reference to Asperger’s alleged protection of children (accessed 5 July 2017).

7 Johns Hopkins University Library holds the journal in which Asperger published his 1938 paper, covering the years 1888 to 1940 (and then again from 1947), https://catalyst.library.jhu.edu/catalog/bib_13574 (accessed 28 July 2016). The 1938 volume has a notation on the back cover that the copy was indeed received in 1938. Personal correspondence with Daniel Bell (Welch Medical Library, Johns Hopkins University), 1 August 2016. For a defense of Kanner’s record, see [107]. For an interesting (if in some elements speculative) discussion of Frankl’s role in the history of autism, see [108].

8 I decided to share results of my work with the authors after meeting with John Donvan in September 2014 in Washington, D.C. He had contacted me after discovering my work via reports in Austrian media [109]. To his credit, Silberman quotes the new evidence against Asperger in the recent editions of his book.

9 Municipal and Provincial Archives Vienna (WStLA), 1.3.2.209.1.A47, Kinderklinik; Heilpädagogische Station: Krankengeschichten. The files were found at the pediatric clinic and transferred to the archive in 2004 [77].

10 In 1935, an American visitor described the ward as “unique” and “the only one of its kind in Europe.” He was struck above all by the methodological and theoretical eclecticism and “an appreciable absence of what are ordinarily regarded as rigid methods, apparatus, statistics, formulae and slogans,” a “highly subjective approach” which in his eyes set the clinic apart from contemporary scientific standards ([110]: 266, 274). The paper was written before Asperger took over the ward in May 1935, and it provides a vivid description of the unusual methods that were part of Lazar’s legacy.

11 WStLA, 1.3.2.202, Personalakt Hans Asperger, Asperger to Magistratsdirektion Wien, 16 September 1936, Beilage: Lebenslauf.

12 The second one would appear in 1937 [71] (not to be confused with the paper of the same title he published in 1938, in which he first described a case of “autistic psychopathy” [1]).

13 DÖW 6217, Liste von Gelehrten österreichischen Ursprungs in den Vereinigten Staaten, 1958; ([22]: 87). Frankl obtained his medical degree in 1922 and joined the Vienna Medical Chamber in 1924 (WStLA, 1.3.2.212.K1 Ärzte: Georg Frankl); it is unknown what he did between 1922 and 1927. Frankl’s publications show that he was an established scholar of Heilpädagogik before Asperger (e.g., [111–116]).

14 A list of scholars of Austrian origin compiled after the war by Austrian authorities lists him as “George Frankl, b. 1897; Child Psychiatrist, Kansas Univ. Medical Center, Kansas City.” DÖW 6217, Liste von Gelehrten österreichischen Ursprungs in den Vereinigten Staaten, 1958.

15 “Anni Frankl, born 25 April 1897; Asst. Prof. of Psychology; University of Kansas, Lawrence, Kansas.” DÖW 6217, Liste von Gelehrten österreichischen Ursprungs in den Vereinigten Staaten, 1958.

16 Asperger in his later years suggested that he was a direct successor to Erwin Lazar, while in reality, it was Bruck who introduced him to the therapeutic pedagogy as it was practiced at the Viennese clinic ([4]: 118–9).

17 Since he never joined the NSDAP, in 1945, Asperger was considered politically “not incriminated” by the Austrian authorities and not affected by denazification measures. For details, see the “Asperger in the post-war years” section.

18 WStLA, 1.3.2.202, Personalakt Erwin Jekelius (cf. [117]: 114); Jekelius published one paper on the ward’s patients [118].

19 Universitätsarchiv Wien, MED PA 17, Personalakt Hans Asperger, “Curriculum vitae,” undated.

20 Eppinger commited suicide in 1946, presumably because he feared being indicted in Nuremberg for his involvement in medical experiments at the Dachau concentration camp [119]. Asperger rarely published with co-authors; between 1930 and 1952, apart from Risak, he collaborated with Josef Feldner (whom he considered his teacher at the clinic) and three colleagues from the circle of Hamburger’s assistants.

21 Much less is known about the group of trainee doctors and assistants (before obtaining their Habilitation), such as Frankl and Asperger. It is unknown if any Jewish assistants remained after Frankl’s departure in 1937.

22 Asperger joined the CDSB in 1921 or 1922: Michael Hubenstorf, Hans Asperger–Biography (unpublished manuscript). In his 1974 interview, Asperger described how the Bund influenced his experience of puberty, which also points to the early 1920s. Overall, his account of the Bund was a-political.

23 According to another source, the Bund had 1200 members in 1937 ([36]:590).

24 See for example Bund Neuland’s periodical *Neue Jugend*, 1935 to 1937.

25 Taras Borodajkewycz, whose anti-Semitic remarks in 1965 triggered an affair regarding persistent (neo-)Nazi sentiments in Austria, had been another prominent Nazi-Catholic “bridge-builder” with close ties to Neuland ([34]: 207–8).

26 One example, “Metropolitan civilization was to 80% a Jewish product, and its power over Germany was nearly absolute. This state of affairs had to be ended in a quick and radical way.” ([120]: 93)

27 For an analysis of the Bund’s heterogeneous ideology and the relationship between its “right” and “left” wing, see [36]: 807–81.

28 WStLA, 1.3.2.202, Personalakt Hans Asperger, Fragebogen, 7 Oct. 1940.

29 WStLA, 1.3.2.202, Personalakt Hans Asperger, Fragebogen, 7 Oct. 1940.

30 WStLA, 1.3.2.202, Personalakt Hans Asperger, Fragebogen, 7 Oct. 1940. Another entry refers to Asperger’s membership in the Vaterländische Front (Fatherland Front, since 1934), the single party of the Austrofascist regime that governed Austria from 1933/34 to 1938. Since membership was obligatory for public employees, however, this is not a reliable indicator of political sympathies.

31 One of these common elements was anti-Semitism, in its religious or racist version. The few available documents on Asperger’s attitude towards Jews (including Jewish patients) are analyzed in the “Asperger’s Jewish patients” section.

32 In German, the passage reads: “Die nationalsozialistische Zeit kam, wobei es aus meinem bisherigen Leben klar war, dass man wohl mit vielen sagen wir nationalen in Anführungszeichen Dingen mitkam, aber nicht mit den Unmenschlichkeiten.” Here as elsewhere in this paper, the translation reflects Asperger’s often convoluted and ambiguous style in order to allow readers to form their own opinion.

33 Private Archive Maria Asperger Felder, Hans Asperger, travel diary, spring 1934. Cited in [10]:103. I asked Asperger Felder for any relevant documents from her father (particularly, any evidence for his alleged troubles with the regime), but she declined, citing fears that Asperger’s statements from the period might be misconstrued (personal correspondence with the author, 29 January 2017).

34 The entire interview lasts 50 min, 3.5 of which are dedicated to the Nazi period and World War II. The translation is quoted from [20]: 18. The passage of his inaugural speech reads: “The connection we had formed during numerous ‘peripatetic’ conversations was so strong that he [Hamburger] felt impelled to save me twice from being arrested by the Gestapo with personal commitment and risking considerable danger to himself, although he knew very well that my ideological and political convictions ran counter to his own. I will not forget this!” ([48]: 937; translation by the author). As far as can be inferred from the written record, Hamburger after 1945 never used this alleged episode in his defense, although he faced considerable pressure due to his Nazi past. After Hamburg’s death in 1954, Asperger abstained from writing an obituary for the man who supposedly saved him twice. 1962 is the first known mention of the alleged Gestapo episode and the only one where Asperger explicitly speaks of the possibility of arrest.

35 For a review of Asperger’s post-war reflections on National Socialism, see the final section of this paper.

36 On 18 August 1939, the Reich Ministry of the Interior ordered the compulsory registration of “malformed etc. children.” Notifications were collected by the public health offices and forwarded to the Reich Committee tasked with the implementation of the child “euthanasia” program: WStLA, 1.3.2.213.A1.155.150.30b,c, RdErl.d.RMdI. IVb 3.088/39-1079/Mi, 18 August 1939. Under the Law for the Prevention of Hereditarily Diseased Offspring of July 1933, specially created Hereditary Health Courts could mandate sterilization in case of one of the following diagnoses: congenital feeblemindedness, schizophrenia, manic-depressive disorders, hereditary epilepsy, Huntington’s chorea, hereditary deafness or blindness, severe physical deformations, and severe alcoholism [61].

37 Austrian State Archive (ÖStA), AdR, Gauakt 36055 Hans Asperger, NSDAP, Gauleitung Wien to Franz Hamburger, 13 July 1940.

38 WStLA, 1.3.2.202, Personalakt Hans Asperger, Geheime Staatspolizei, Staatspolizeileitstelle Wien to Personalamt Wiener Gemeindeverwaltung, 9 November 1940.

39 ÖStA, AdR, Gauakt 36055 Hans Asperger, STK/I-S-9733, Staatskommissar Otto Wächter an das ehemalige Unterrichtsministerium, 7 June 1939.

40 ÖStA, AdR, Gauakt 36055 Hans Asperger, III D U/R/G1, Gaupersonalamt, 5 January 1939: Asperger Dr. Johann, signature illegible.

41 We would additionally have to explain why Franz Hamburger also kept silent over an episode that could have helped his reputation, which after the war was badly damaged by his heavy Nazi involvement. Wolfgang Neugebauer, one of the leading historians of National Socialism in Austria, considers the Gestapo episode a complete fabrication (personal correspondence with the author, 2 December 2016).

42 Hansi Busztin, Eitelkeit, undated [unpublished manuscript, mid to late 1980s]. Personal correspondence with Anna Goldenberg (Hans Busztin’s granddaughter), 22 January 2017.

43 WStLA, 1.3.2.202, Personalakt Hans Asperger, Hauptgesundheitsamt to Hauptpersonalamt, 28 June 1943.

44 ÖStA, AdR, Gauakt 36055 Hans Asperger.

45 WStLA, 1.3.2.202, Personalakt Hans Asperger, Fragebogen, 7 October 1940.

46 In Vienna, 31.4% of those physicians who remained after the exclusion of all Jews from the profession joined the Nazi party, but only 17.6% were also members of the Ärztebund*.* In all of Austria (no numbers available for Vienna only), a further 8.5% became Ärztebund candidates like Asperger: Hubenstorf M. Nazi Doctors in Vienna (paper given at the conference “Austrian Physicians and National Socialism,” Vienna, 16 April 2015).

47 ÖStA, AdR, Gauakt 36055 Hans Asperger, Kreisleitung I. der NSDAP Wien to Ortsgruppenleiter, Fragebogen zur politischen Beurteilung, 6 December 1938 (filled in and signed on 4 and 23 January 1939).

48 ÖStA, AdR, Gauakt 36055 Hans Asperger, Gaupersonalamtsleiter Stowasser, 1 November 1940.

49 ÖStA, AdR, Gauakt 36055 Hans Asperger, Schreiben des Führers des SD-Leitabschnittes Wien to Personalamt der Stadt Wien, 14 November 1940.

50 WStLA, 1.3.2.202, Personalakt Hans Asperger, Dozentenführer der Universität (Dr. Marchet) to Dekanat, 17 November 1942 and 15 April 1943.

51 UAW, MED PA 17, Personalakt Hans Asperger, “Bestätigung,” 22 May 1942. The procedure to officially obtain the title was suspended because of the war.

52 Private Archive Maria Asperger Felder, Josef Feldner, letter, undated. Cited in [10]: 104.

53 WStLA, 1.3.2.209.1.A47, Kinderklinik; Heilpädagogische Station: Krankengeschichten.

54 WStLA, 1.3.2.209.1.A47, Kinderklinik; Heilpädagogische Station: Krankengeschichten 1938.

55 The city had approximately 200 Jewish foster children in its charge; how many of these were living with non-Jewish foster parents is unknown.

56 WStLA, 1.3.2.209.1.A47, Kinderklinik; Heilpädagogische Station: Krankengeschichten 1938, Alfred S.; 1.3.2.209.1.A57, Psychiatrie: Krankengeschichten: Männer, Alfred S. According to Austrian data protection laws, in many cases (such as documents from public archives), full names can only be published if the person in question is confirmed dead.

57 WStLA, 1.3.2.209.1.A47, Kinderklinik; Heilpädagogische Station: Krankengeschichten 1938, Walter Brucker.

58 WStLA, 1.3.2.209.1.A47, Kinderklinik; Heilpädagogische Station: Krankengeschichten 1938, Ivo P. As in many other cases after March 1938, Asperger closed his report on Ivo with the salutation “Heil Hitler.” Asperger exhibited a similar detachment in many of his recommendations for the transferal of children to Spiegelgrund, to which he referred as though to a normal pedagogical institution, despite its function as a killing facility. For an analysis of these transferals, see the “Limits of ‘educability’: Asperger and the Spiegelgrund ‘euthanasia’ facility” and “Asperger’s diagnoses compared to those at Spiegelgrund” sections. Ivo P. was not sent to a “normal” home, as Asperger had recommended, but to an institution for “non-Aryan” children. His trace in the youth welfare office’s records ends in 1943: 1.3.2.207.A6, KÜST-Kartei, Ivo P.

59 WStLA, 1.3.2.209.1.A47, Kinderklinik; Heilpädagogische Station: Krankengeschichten 1939, Marie Klein.

60 WStLA, 1.3.2.212, A 5/4, Gundel to Scharizer (Stellvertretender Gauleiter), 3 April 1941.

61 WStLA, 1.3.2.209.1.A47, Kinderklinik; Heilpädagogische Station: Krankengeschichten 1939, Marie Klein.

62 WStLA, 1.3.2.209.1.A47, Kinderklinik; Heilpädagogische Station: Krankengeschichten 1939, Lizzy Hofbauer. In February 1941, she was a patient at the Vienna Jewish Hospital on the ward of Viktor Frankl (1905–1997), who after the war would rise to worldwide fame as the author of the best-selling book *Man’s Search for Meaning* [121] and as the creator of logotherapy and existential analysis. Citing “lack of space,” Frankl wrote to Otto Pötzl (1877–1962) to have Lizzy transferred to the latter’s Psychiatric-Neurological University Clinic, from where she was sent to Steinhof Psychiatric Hospital. Ten days after Frankl’s letter, Lizzy was put on a transport to the extermination center Hartheim near Linz, run by the “T4” euthanasia program: WStLA, 1.3.2.209.1.A56, Krankengeschichte Lizzy Hofbauer; WStLA, 1.3.2.209.2.B5, Standesprotokoll 1941. This case stands in contrast to Frankl’s later claim that Pötzl and himself had worked together to transfer Jewish patients from Pötzl’s Psychiatric University Clinic to the Jewish hospital in order to save them from “euthanasia” ([122]: 60–1).

63 For a history of anti-Semitism in Austria, see [123].

64 The total number of forced sterilizations between 1934 and 1945 is estimated at 400,000 ([60]: 238).

65 WStLA, 1.3.2.212.A7/7, “Richtlinien für die Beurteilung der Erbgesundheit,” circular of the Reich Minister of the Interior (18 July 1940)-IV b 1446/40-1072c.

66 The author also shows how educators frequently tried to improve their professional standing by stressing the potential of their own clientele, often at the expense of other groups labeled as farther down the value hierarchy (a strategy he calls “demarcation towards below”). On the accommodation of Heilpädagogik as a whole with the Nazi regime, see above.

67 WStLA, 1.3.2.209.1.A47, Kinderklinik; Heilpädagogische Station: Krankengeschichten, e.g., case histories Elfriede P. (1935), who according to Asperger had shown a suspicious “interest” when sexually abused at age 5, or Kurt K. (1936), whom Asperger labeled an “unstable criminal” at the age of 12, see also the “Asperger in the post-war years” section.

68 This view has recently been challenged on the grounds that the adoption of electroconvulsive therapy in Nazi Germany was in fact slower than previously assumed [124, 125]. My argument in this context, however, is based on the interest the “T4” organization took in the issue, which is not in dispute.

69 In 1941, Jekelius fell out of favor with Hitler because of an affair with his sister Paula Hitler, who lived in Vienna. Jekelius was drafted into the army and sent to the front. After the war, Jekelius was arrested and put on trial by the Soviets. He died in a Moscow prison in 1952. Ministry of State Security of the USSR, Interrogation transcript with inmate Erwin Jekelius, 9 July 1948 (copy and German translation in DÖW 51401). For more details on Jekelius, see [13]. The “Action T4” was stopped in August 1941 after protests from victims’ relatives and the churches. Nevertheless, in many psychiatric hospitals, patients continued to be killed until 1945 by means of starvation, neglect, and frequently also by more direct means such as poisoning or electric shock [80, 126].

70 WStLA, 1.3.2.202, Personalakt Hans Asperger, Fragebogen für den Personalkataster, 27 November 1940, and further documents in the file.

71 Jekelius did not explicitly mention the “euthanasia” killings—their existence was a state secret—but the meaning would have been clear to professional insiders (for more on how the knowledge of the killings spread among the population, see the “Limits of ‘educability’: Asperger and the Spiegelgrund ‘euthanasia’ facility” section). In 1942, the Spiegelgrund “euthanasia” clinic was renamed and for a period had the term “curative pedagogy” in its official designation—an indication that the discipline, far from being under threat from the regime, at least for a time was actually gaining ground; WStLA, 1.3.2.209.A1, Anstaltenamt, Normalien des Anstaltenamtes der Hauptabteilung V, A Nr. 500, 23 November 1942.

72 In this text, Asperger refers to children “whose abnormity is not of a type that would call for sterilization, who would socially fail without our understanding and guiding assistance, but who with this help are able to occupy their place in the large organism of our people.” [“Mein Augenmerk soll aber heute vor allem auf die gerichtet sein, deren Abnormität nicht von der Art ist, daß eine Sterilisierung in Frage käme, die aber auch ohne unsere verstehende und führende Hilfe sozial scheitern würden, mit dieser Hilfe aber ihren Platz in dem großen Organismus des Volkes ausfüllen können.”] These children had little to do with those targeted for child ‘euthanasia’ or forced sterilization. Other examples for Asperger’s utilitarian argumentation in favor of Heilpädagogik are to be found in ([63]:946) or ([2]:135). Arguing that children with abnormalities needed special assistance in order to become useful members of society (including the military) was a common strategy of professionals in Heilpädagogik and related fields during National Socialism, see for example ([64]:178–92).

73 WStLA, 1.3.2.213.A1.158.160.30, Tagesordnung der vom 31. August bis 4. September 1940 in Wien stattfindenden 47. Ordentlichen Tagung der Deutschen Gesellschaft für Kinderheilkunde. Conti hanged himself in 1945 in a prison cell in Nuremberg.

74 At the conference, the German Association for Youth Psychiatry and Curative Pedagogy was established, additional proof that the latter discipline was not controversial in the eyes of the regime [127]. For the proceedings of the 1940 Vienna conference, see [72].

75 Between 1950 and 1986, 6459 children were admitted to the Heilpädagogik ward; of these, 228 (3.5%) were diagnosed with autistic spectrum disorders (74 “autistic psychopaths,” 83 cases of early infantile autism, 71 cases with autistic features) ([128]: 293).

76 The patient registry of Asperger’s ward contains information on close to 2700 children admitted between 1935 and 1949. The most common reason for admission was “education problems” (*Erziehungsschwierigkeiten*): WStLA, 209.1.B1018 (prov.), Kinderklinik, Index 1921–1964.

77 Heller’s speech also contained powerful arguments against the forced sterilization program that was already underway in Germany. Due to his Jewish background, Heller became an early victim of Nazi persecution after the Anschluss. He died in December 1938, months after a suicide attempt he never recovered from. His wife and daughter were later deported and killed in Riga [129]. *Dementia infantilis Heller* (in English: Heller’s syndrome, now better known as childhood disintegrative disorder) was named after him.

78 Asperger himself did not give a talk at this one-day symposium, but he spoke at the pediatric conference that provided the framework (see above).

79 In Vienna, 96 of approximately 110 pediatricians were considered Jewish according to Nazi racial legislation and lost their livelihoods, their homes, and in at least 12 cases their lives ([130]: 756).

80 UAW, MED PA 17, Personalakt Hans Asperger, Prof. Franz Hamburger, Referaterstattung zur Habilitationsarbeit des Dr. Hans Asperger, 28 December 1942.

81 WStLA, 1.3.2.202, Personalakt Hans Asperger, Personalamt to Leiter Personalamt, 9 November 1940; Gemeindeverwaltung der Stadt Wien to Hans Asperger, 12 November 1940. Asperger had worked part-time for the city’s child welfare program in addition to his position at the Pediatric University Clinic since 1936.

82 UAW, MED PA 17, Personalakt Hans Asperger, Prof. Franz Hamburger, Referaterstattung zur Habilitationsarbeit des Dr. Hans Asperger, 28 December 1942. After 1938, the NSV took over numerous children’s homes; racially or politically “undesirable” children were removed from these institutions and concentrated in facilities such as Spiegelgrund: WStLA, 1.3.2.212.A7, Parville to Vellguth et al., 23 June 1941; Erlass Vellguth, 5 July 1942.

83 In Vienna, information on more than 767,000 individuals was collected in this context. For details on this so-called *erbbiologische Bestandsaufnahme* (hereditary inventory), see [131].

84 WStLA, 1.3.2.209.10.A1 (Spiegelgrund), Krankengeschichten: überlebende Knaben und Mädchen 1941–1945.

85 WStLA, 1.3.2.209.1.A47, Kinderklinik; Heilpädagogische Station: Krankengeschichten.

86 WStLA, 1.3.2.209.1.A47, Kinderklinik; Heilpädagogische Station: Krankengeschichten 1912–1944, case histories Gertrude S., Hildegard S., Helmuth R., Theodor M., Eduard G. (1940 and 1941), Elfriede V. (filed under 1932, documents from 1942), and Ernst T. (filed under 1935, documents from 1942). The Heilpädagogik ward mentioned to the Department of Hereditary and Racial Hygiene that Ernst T.’s father was Jewish, information that put the child in considerable danger. His fate is unknown.

87 WStLA, 1.3.2.209.10, Nervenklinik für Kinder, Krankengeschichten: überlebende Mädchen und Knaben 1941–1945, Adolf R., Otto S., Karl S., Charlotte H. The terms “degenerative” and “degeneration,” unlike today, clearly referred to negative hereditary traits, in the same sense as the now historic German term *Entartung*, see ([132]: 786–7).

88 WStLA, 1.3.2.209.1.A47, Kinderklinik; Heilpädagogische Station: Krankengeschichten 1912–1944.

89 The 1699 surviving Vienna records of initiated sterilization procedures show the following age distribution: 13 years, 1 case; 14 years, 5; 15 years, 22; 16 years, 31; 17 years, 44; 18 years, 76 (WStLA, 2.3.15.A1.1-3, Namensindex, Claudia Spring).

90 Verordnung zur Ausführung des Gesetzes zur Verhütung erbkranken Nachwuchses, 5 December 1933, Reichsgesetzblatt Teil 1, Nr. 138, 1021.

91 WStLA, 1.3.2.209.1.A47, Kinderklinik; Heilpädagogische Station: Krankengeschichten, 15/1933, Therese B.

92 WStLA, 1.3.2.209.1.A47, Kinderklinik; Heilpädagogische Station: Krankengeschichten 1912–1944, case history Ernst M., 1930, Wiener Kinderklinik to Hauptgesundheitsamt, Erb- und Rassenpflege, 2 March 1942.

93 Gesetz zur Verhütung erbkranken Nachwuchses, 25 July 1933, RGBl. I 1933, S. 529-31, §14.

94 DÖW 51983, Stadtschulrat für Wien to Stephan I., 22 December 1939; Rosalia I. to Leo Navratil, 7 January 1962; Susanne Zander to Maria Asperger-Felder, 16 October 2009 (I thank Kathrin Hippler for these documents).

95 DÖW 51983, Stadtschulrat für Wien to Stephan I., 22 December 1939; Rosalia I. to Leo Navratil, 7 January 1962.

96 WStLA, 1.3.2.209.10, Nervenklinik für Kinder, Krankengeschichten: verstorbene Mädchen und Knaben 1940–1945, Krankengeschichte Herta Schreiber, Heilpädagogische Abteilung der Universitäts-Kinderklinik Wien, Befund Herta Schreiber, 27 June 1941, gez. Dr. Asperger. Overall, files pertaining to 562 out of a total of 789 victims have been preserved.

97 WStLA, 1.3.2.209.10, Nervenklinik für Kinder, Krankengeschichten: verstorbene Mädchen und Knaben 1940–1945, Krankengeschichte Herta Schreiber. Together with hundreds of body parts from other children killed at Spiegelgrund, Herta’s brain was preserved and used for research until decades after the war. The human remains were buried in 2002 in Vienna (cf. [85]).

98 WStLA, 1.3.2.209.10, Nervenklinik für Kinder, Krankengeschichten: verstorbene Mädchen und Knaben 1940–1945, Krankengeschichte Herta Schreiber, Einlagebogen, 13 July 1941, no signature.

99 The infamous “euthanasia” propaganda film *Ich klage an* (I Accuse) in a subplot uses a girl with encephalitis-induced brain damage to justify the killing of mentally disabled children.

100 WStLA, 1.3.2.209.10, Nervenklinik für Kinder, Krankengeschichten: verstorbene Mädchen und Knaben 1940–1945, Krankengeschichte Elisabeth Schreiber (born 9 October 1936), Abschrift des Gutachtens der Univ. Kinderklinik Wien, 27 October 1941, Dr. Asperger.

101 WStLA, 1.3.2.209.10, Nervenklinik für Kinder, Krankengeschichten: verstorbene Mädchen und Knaben 1940–1945, Krankengeschichte Elisabeth Schreiber.

102 DÖW E 18.282, Vernehmung des Beschuldigten Dr. Illing, 22 October 1945, cited in ([133]: 44).

103 Ministerium für Staatssicherheit der UdSSR, Verhörprotokoll des Inhaftierten Erwin Jekelius, 9 July 1948, Blatt 48/11f. (copy and translation in DÖW 51401), also ([134]: 208–9).

104 DÖW 7906, Luftpost, Royal Air Force, fall 1941.

105 WStLA, 1.3.2.209.10, Nervenklinik für Kinder, Krankengeschichten: verstorbene Mädchen und Knaben 1940–1945, Krankengeschichte Ulrike Mayerhofer, Krankengeschichte Richard Draskovic.

106 In this case, Asperger used the term “autistic” in its original Bleulerian sense of a symptom associated with schizophrenia.

107 In the Gugging psychiatric hospital, hundreds of patients were killed by doctors and nurses during the war [135].

108 Lower Austria Provincial Archives (NÖLA), Erlass der Reichsstatthalterei in Niederdonau (Gauselbstverwaltung), 9 December 1941.

109 NÖLA, Reichsstatthalter in Niederdonau to Dezernat III b-2, 2 March 1942.

110 NÖLA, Reichsstatthalter in Niederdonau to Dezernat III b-2, 2 March 1942.

111 WStLA, 1.3.2.209.10, Nervenklinik für Kinder, B 4 Totenbuch.

112 NÖLA, Reichsstatthalter in Niederdonau to Dezernat III b-2, 2 March 1942.

113 WStLA, 1.3.2.209.10, Nervenklinik für Kinder, B 4 Totenbuch.

114 NÖLA, Heil- und Pflegeanstalt Gugging, Krankengeschichte Engelbert Deimbacher.

115 NÖLA, Heil- und Pflegeanstalt Gugging, Krankengeschichte Georgine Schwab.

116 Not all the files are preserved, and the surviving ones may not be complete. In a sample of 76 files concerning children who died at Spiegelgrund or, in a few cases, at Gugging, 21 contain correspondence with parents. In 6 of these (including the ones quoted above), the parents had asked to take the children into their care (NÖLA, Heil- und Pflegeanstalt Gugging, Krankengeschichten der Kinderanstalt).

117 The 30 cases in the sample with detailed documentation: WStLA, 1.3.2.209.10.A1 (Spiegelgrund), Krankengeschichten: überlebende Knaben und Mädchen 1941–1945, Leo A., Karl E., Walter G., Arnold H., Alfred H., Johann K., Franz K., Gottfried K., Rudolf N., Roman R., Johann R., Adolf R., Josef R., Otto S., Karl S., Gerald St., Rudolf St., Richard S., Johann T., Leopold V., Erich We., Erich Wi., Charlotte H., Edith H., Lucia K., Edith M., Charlotte M., Hildegard P., Helene P., Charlotte R. The 10 cases excluded from the direct comparison between Spiegelgrund and Asperger’s ward due to insufficient documentation (but included in the sample of 40): Anton R., Walter S., Erwin T., Mathilde H., Herta P., Margarete Schw., Margarethe Sch., Friedrich K., Theodor P. and Robert Sch.; the last three belong to WStLA, 1.3.2.209.1.A47, Kinderklinik; Heilpädagogische Station: Krankengeschichten.

118 As mentioned, Spiegelgrund’s first director had trained at the Heilpädagogik ward; moreover, Spiegelgrund was the only institution in the area that at least for a time in 1942 had the term “Heilpädagogik” in its name ([13]: 201).

119 Survivors’ testimonies can be found at [http://gedenkstaettesteinhof.at/de/interviews, accessed 23 November 2016].

120 Friedrich K. was just under 8 years old when in March 1942 Asperger diagnosed him with an “autistic personality” and a “severe psychopathic state”; according to Asperger, the boy was “intellectually low-ranking” but sometimes “surprised with good performances in some areas.” Due to his “irresponsible” and unmotivated “compulsive activity” and his “substantial mental abnormality,” Asperger considered the Spiegelgrund institution the only suitable option: WStLA, 1.3.2.209.1.A47, Kinderklinik; Heilpädagogische Station: Krankengeschichten, 1942, Friedrich K. The boy was transferred to Spiegelgrund in May 1942 (via *Kinderübernahmsstelle*), where he stayed for 10 months before being sent to the children’s home Wimmersdorf: WStLA, 1.3.2.207.A6, index card Friedrich K.

121 WStLA, 1.3.2.209.10.A1, Krankengeschichten: überlebende Knaben und Mädchen 1941–1945, Gerald St., Asperger to BJA III, 30 July 1941.

122 WStLA, 1.3.2.209.10.A1, Krankengeschichten: überlebende Knaben und Mädchen 1941–1945, Gerald St., 1. Psychologisches Gutachten, 11 June 1942, signed Igor Caruso. After 1945, Caruso (1914–1981) rose to prominence in Austria as the founder of his own psychoanalytical school. His involvement at Spiegelgrund became the subject of heated debate following a 2008 publication in which the topic was raised for the first time [136].

123 WStLA, 1.3.2.209.10.A1, Krankengeschichten: überlebende Knaben und Mädchen 1941–1945, Gerald St., Ärztliches Gutachten, 22 July 1942, signed Heinrich Gross and Ernst Illing. After 1945, Heinrich Gross (1915–2005) became one of the most prominent psychiatrists in Austria, partly thanks to the scientific exploitation of the brain specimens obtained from the children killed at Spiegelgrund ([85]:112–4).

124 WStLA, 1.3.2.209.10, Nervenklinik für Kinder, Krankengeschichten: überlebende Mädchen und Knaben 1941–1945, Leo A., Befund, 27 December 1940, signed Asperger.

125 WStLA, 1.3.2.209.10, Nervenklinik für Kinder, Krankengeschichten: überlebende Mädchen und Knaben 1941–1945, Leo A., Befund und Gutachten, 23 September 1941, signed Heinrich Gross and Erwin Jekelius. It should be noted that the boy returned both to Spiegelgrund and the Heilpädagogik ward in 1942/1943, but the documents pertaining to these stays (assessments by Ernst Illing, Jekelius’s successor at Spiegelgrund, and by one of Asperger’s colleagues) are not relevant here.

126 WStLA, 1.3.2.209.10, Nervenklinik für Kinder, Krankengeschichten: überlebende Mädchen und Knaben 1941–1945, Karl E., Befund, 25 October 1940, signed Asperger.

127 WStLA, 1.3.2.209.10, Nervenklinik für Kinder, Krankengeschichten: überlebende Mädchen und Knaben 1941–1945, Karl E., Führungsbericht und Gutachten, signed Margarete Hübsch and Erwin Jekelius, 19 September 1941.

128 WStLA, 1.3.2.209.10, Nervenklinik für Kinder, Krankengeschichten: überlebende Mädchen und Knaben 1941–1945, Johann K., Befund, signed Asperger, 16 September 1942.

129 WStLA, 1.3.2.209.10, Nervenklinik für Kinder, Krankengeschichten: überlebende Mädchen und Knaben 1941–1945, Johann K., Gutachterliche Äußerung, signed Ernst Illing, 6 January 1943.

130 WStLA, 1.3.2.209.10, Nervenklinik für Kinder, Krankengeschichten: überlebende Mädchen und Knaben 1941–1945, Charlotte H., Heilpädagogisches Gutachten, signed Asperger, 20 February 1941.

131 WStLA, 1.3.2.209.10, Nervenklinik für Kinder, Krankengeschichten: überlebende Mädchen und Knaben 1941–1945, Charlotte H., Gutachterliche Äußerung, signed Ernst Illing, 5 August 1942.

132 WStLA, 1.3.2.209.10.A1, Krankengeschichten: überlebende Knaben und Mädchen 1941–1945, Johann T., Asperger to Wohlfahrtsamt der Bezirkshauptmannschaft Mödling-Liesing, 26 November 1938.

133 WStLA, 1.3.2.209.10.A1, Krankengeschichten: überlebende Knaben und Mädchen 1941–1945, Johann T., “Abschrift Küst” on Johann T., Befund Jekelius, 9 May 1941.

134 WStLA, 1.3.2.209.10.A1, Krankengeschichten: überlebende Knaben und Mädchen 1941–1945, Hildegard P., Heilpädagogisches Gutachten, signed Asperger, 2 October 1940.

135 WStLA, 1.3.2.209.10, Nervenklinik für Kinder, Krankengeschichten: überlebende Mädchen und Knaben 1941–1945, Hildegard P., Führungsbericht und Gutachten, signed Jokl and Jekelius, 29 May 1941.

136 WStLA, 1.3.2.209.1.A47, Kinderklinik; Heilpädagogische Station: Krankengeschichten, case history Karl M. On the *Wanderhof Herzogsägmühle*, see ([137]: especially 53–4).

137 WStLA, 1.3.2.209.1.A47, Kinderklinik; Heilpädagogische Station: Krankengeschichten.

138 Deutsche Dienststelle, Auskunft zur Wehrmachtdienstzeit des Dr. Hans Asperger, 28 July 2015. He returned to Vienna on 22 September 1945, after several months as a prisoner of war: WStLA, 1.3.2.202, Personalakt Hans Asperger, Inhaltsverzeichnis.

139 The choice of the expression *niederschießen* (to gun down) in what sounds like a carefully scripted monologue hints at the particular character of the warfare he experienced, since it suggests the killing of unarmed individuals rather than military battle. A remark in his textbook, first published in 1952, points in a similar direction: “Also during the war, one had to experience time and again how strong the pressure of the ‘collective’ could be, what terrible deeds a group of people as a whole were capable of, deeds that none of them would have committed on their own in their civil lives” ([130]: 81).

140 WStLA, 1.3.2.202, Personalakt Hans Asperger, Fragebogen, 7 October 1940.

141 UAW, MED PA 17, Personalakt Hans Asperger, Personalblatt, 1 September 1945; Asperger to Professorenkollegium der med. Fakultät, 1 September 1945. On the denazification of the medical profession in Austria, see [138].

142 UAW, MED PA 17, Personalakt Hans Asperger.

143 This conflict also had political overtones, since the dominating figure in post-World War II youth psychiatry was Walter Spiel, a Social Democrat [74].

144 This passage remained in the book at least until the fourth edition, which was published in 1965. The author wishes to thank Thomas Mayer for the reference to this passage. Regarding child “euthanasia,” Asperger declared his fundamental opposition to the idea of “unworthy life” in a letter to a board member of the German Pediatric Association in 1961, when the organization was mired in controversy over the continued membership of Werner Catel, one of three “experts” of the child “euthanasia” program ([139]: 115).

145 Asperger still argued along similar lines in the fourth edition of his handbook on Heilpädagogik ([140]: 284–6).

146 According to a former collaborator, in the relative privacy of a case conference he called a 6-year-old girl a “whore” ([141]: 141). This is in keeping with the fact that in his textbook, Asperger quoted Lambroso and his idea of the “born criminal” and the “born prostitute” ([76]: 85).

147 WStLA, 1.3.2.209.10.A1 (Spiegelgrund), Krankengeschichten: überlebende Knaben und Mädchen 1941–1945, Edith H.

148 The concept of “pension neurosis” was created by German psychiatrists after World War I. It served to deny benefits to war veterans who suffered from what today would be called PTSD. After World War II, the concept was often turned against survivors of Nazi medical experiments, concentration camps, and ghettoes, again by denying a causal link between experiences of persecution and long-term effects on mental health ([142]: 166). A paper that Asperger published in 1939 with his colleague Heribert Goll demonstrates to what extent Asperger thought that inborn (if not necessarily hereditary) characteristics determined personality traits in later life. The article was published in the journal *Der Erbarzt*, edited by leading race hygienist Otmar von Verschuer (1896–1969) [143].

149 WStLA, 1.3.2.207.A1-Zöglingsakten, F-H, 1946. I thank Gertrude Czipke for these documents. On the concept of the “epileptoid personality,” see ([76]: 132–4). There is no indication that Max actually suffered from epilepsy. Asperger’s diagnosis was based purely on personality traits he observed during his interaction with the boy, which he considered typical for epileptics.

150 In 2014, the City of Vienna commissioned a research project on this affair, the results of which were published in 2017 [104].

151 The other Austrian physician whom Kondziella places in this category is Andreas Rett (1924–1997), discoverer of Rett syndrome, a former NSDAP member who became a leading figure in Austrian child neurology after World War II with a special focus on the care of children with disabilities, for whom he proved an effective and tireless advocate. His paternalistic approach, focus on segregation, use of non-approved drugs, and support for sterilization remain controversial [104, 144]. Since Rett was born 18 years after Asperger, the two cases are however difficult to compare with regards to the Nazi period.
