# Correction to: Hans Asperger, National Socialism, and “race hygiene” in Nazi-era Vienna

**DOI:** 10.1186/s13229-021-00433-x

**Published:** 2021-06-21

**Authors:** Herwig Czech

**Affiliations:** Ethics, Collections, and History of Medicine, Medical University of Vienna, Währinger Straße 25, 1090 Vienna, Austria

## Correction to: Molecular Autism (2018) 9:29 10.1186/s13229-018-0208-6

Following publication of the original article [1], the author notified about some errors in the article text, footnotes and references. The corrected parts should be as follows:

Founded in 1921, the Austria-based Bund was a split-off from the Christian-German Student Union (CDSB) but stressed its affinities with the German Youth Movement as represented by the “Meißner formula,” which Asperger cited in 1974 as a guiding principle in his life [3].^21^

Asperger’s political socialization in Neuland likely blinded him to National Socialism’s destructive character due to an affinity with core ideological elements (see [36]: 848–9).^30^

This passage has been quoted as evidence that Asperger tried to publicly protect his patients from forced sterilizations ([8]: 464; more cautiously [9]: 206–7).

This includes Asperger’s mentor Franz Hamburger and also applies to Erwin Jekelius, a youth psychiatrist trained at Hamburger’s clinic, who in 1940 became the main organizer of the “T4” killing operation in Vienna.

Jekelius had received part of his training at the Therapeutic Pedagogy Ward under Asperger’s direction, where he was employed from August 1933 to February 1936 ([10]: 102, [118]).

From Herta’s religious denomination gottgläubig (theistic), it can be inferred that the family had left the Catholic Church under the influence of the Nazis’ opposition to organized religion, a practice that was usually followed only by a radical minority of Nazi sympathizers ([82]: 281–3).

Anny Wödl, a Viennese nurse, had no doubt that the transfer of her son Alfred to Spiegelgrund, enforced in early 1941 despite her resolute resistance, would mean his death ([87]: 298).

In November 1941, the authorities in Niederdonau (the province surrounding Vienna) noticed that patients in the children’s ward at the Gugging psychiatric hospital were not attending school, despite not having been excused.^106^

In this context, a critical assessment of Asperger’s brand of Heilpädagogik with its “pronounced dominance of restrictive pedagogical concepts” ([74]: 613) is overdue.

^1^A copy of the postdoctoral thesis as submitted in 1942 is at the Vienna University Library. It is practically identical to the 1944 paper.

^12^DÖW 6217, Liste von Gelehrten österreichischen Ursprungs in den Vereinigten Staaten, 1958; ([21]: 87).

^29^WStLA, 1.3.2.202, Personalakt Hans Asperger, Fragebogen, 7 Oct. 1940. Another document refers to Asperger’s membership in the Vaterländische Front (Fatherland Front, since 1934), the single party of the Austrofascist regime that governed Austria from 1933/34 to 1938.

^48^WStLA, 1.3.2.202, Personalakt Hans Asperger, Schreiben des Führers des SD-Leitabschnittes Wien to Personalamt der Stadt Wien, 14 November 1940.

^138^The choice of the expression niederschießen (to gun down) in what sounds like a carefully scripted monologue hints at the particular character of the warfare he experienced, since it suggests the killing of unarmed individuals rather than military battle. A remark in his textbook, first published in 1952, points in a similar direction: “Also during the war, one had to experience time and again how strong the pressure of the “collective” could be, what terrible deeds a group of people as a whole were capable of, deeds that none of them would have committed on their own in their civil lives” ([76]: 81).

Abbreviation should be: CDSB: Christian-German Student Union.

Reference 13: Czech H. Der Spiegelgrund-Komplex. Kinderheilkunde, Heilpädagogik, Psychiatrie und Jugendfürsorge im Nationalsozialismus. Österreichische Zeitschrift für Geschichtswissenschaft. 2014;25:194–219.

Reference 40: Korger F. Zur heutigen Behandlung der Judenfrage. Neuland. Blätter jungkatholischer Erneuerungsbewegung. 1933;10:171–4.
